# Molecular basis of dengue virus serotype 2 morphological switch from 29°C to 37°C

**DOI:** 10.1371/journal.ppat.1007996

**Published:** 2019-09-19

**Authors:** Xin-Ni Lim, Chao Shan, Jan K. Marzinek, Hongping Dong, Thiam Seng Ng, Justin S. G. Ooi, Guntur Fibriansah, Jiaqi Wang, Chandra S. Verma, Peter J. Bond, Pei-Yong Shi, Shee-mei Lok

**Affiliations:** 1 Program in Emerging Infectious Diseases, Duke-National University of Singapore Medical School, Singapore, Singapore; 2 Centre for Bioimaging Sciences, National University of Singapore, Singapore, Singapore; 3 Novartis Institute for Tropical Diseases, Singapore, Singapore; 4 Department of Biochemistry and Molecular Biology, University of Texas Medical Branch, Galveston, Texas, United States of America; 5 Bioinformatics Institute, Agency of Science, Technology and Research (A*STAR), Singapore, Singapore; 6 School of Biological Sciences, Nanyang Technological University, Singapore, Singapore; 7 Department of Biological Sciences, National University of Singapore, Singapore, Singapore; 8 Sealy Center for Structural Biology & Molecular Biophysics, University of Texas Medical Branch, Texas, United States of America; The University of Chicago, UNITED STATES

## Abstract

The ability of DENV2 to display different morphologies (hence different antigenic properties) complicates vaccine and therapeutics development. Previous studies showed most strains of laboratory adapted DENV2 particles changed from smooth to “bumpy” surfaced morphology when the temperature is switched from 29°C at 37°C. Here we identified five envelope (E) protein residues different between two alternative passage history DENV2 NGC strains exhibiting smooth or bumpy surface morphologies. Several mutations performed on the smooth DENV2 infectious clone destabilized the surface, as observed by cryoEM. Molecular dynamics simulations demonstrated how chemically subtle substitution at various positions destabilized dimeric interactions between E proteins. In contrast, three out of four DENV2 clinical isolates showed a smooth surface morphology at 37°C, and only at high fever temperature (40°C) did they become “bumpy”. These results imply vaccines should contain particles representing both morphologies. For prophylactic and therapeutic treatments, this study also informs on which types of antibodies should be used at different stages of an infection, i.e., those that bind to monomeric E proteins on the bumpy surface or across multiple E proteins on the smooth surfaced virus.

## Author summary

DENV2 particles have been shown to change their morphologies (compact smooth to loose bumpy surfaced) when temperature is switched from 28°C to 37°C. We used two DENV2 viruses both belonging to the same strain designation but with a different passage history—one of which exhibited the smooth surfaced morphology while the other was bumpy surfaced, observed by cryoEM. We mutated residues in the E protein of the DENV2 infectious clone that has the smooth surfaced morphology to determine if any could result in a bumpy morphology. Results showed several different mutations could lead to this change. Using molecular dynamics simulations, we showed how these mutations likely destabilize the E protein dimeric interactions. We investigated whether the bumpy morphology also occurs in DENV2 clinical isolates, and showed that these viruses can exhibit both morphologies, indicating that vaccine and therapeutics development should target both virus forms.

## Introduction

Dengue virus (DENV) infects ~400 million people annually around the world especially in the tropical and sub-tropical regions [1, 2]. It causes diseases ranging from mild dengue fever to severe dengue hemorrhagic fever (DHF) and dengue shock syndrome (DSS). DENV is a flavivirus, and is transmitted by the mosquito vectors *Aedes aegypti* and *Aedes albopictus*. Other important human pathogens in the *flaviviridae* family are West Nile (WNV), Yellow fever, Japanese encephalitis and Zika virus. DENV comprises of four serotypes (DENV1-4). The sequence variability between the four serotypes is about 25 to 40% and between strains within a serotype is ~3%[3, 4].

The development of vaccines is complicated by the presence of four serotypes. When an individual is infected with one serotype, subsequent infection with another serotype may result in the development of DHF or DSS. This antibody-dependent enhancement (ADE) phenomenon is proposed to be due to antibodies elicited in a primary infection against the first serotype subsequently binding to but not neutralizing the other serotype in the second infection. Non-neutralizing or sub-neutralizing concentrations of antibodies binding to the virus may help to concentrate the virus onto the Fcγ receptor on the surface of monocytes and macrophage cells, thereby leading to an enhancement of infection [5]. This suggests that an effective vaccine should simultaneously stimulate equally strong neutralizing antibody responses against all four serotypes. Currently, several vaccine candidates have been tested in clinical trials. Thus far, CYD-TDV is the only licensed dengue vaccine. However, this tetravalent dengue vaccine showed poor efficacy towards DENV2 and moderate efficacy to DENV1, DENV3 and DENV4 [6, 7]. Interestingly, we previously observed that the DENV2 mature virus structure can be heterogenous at 37°C, which poses an additional complication when designing vaccines.

DENV contains a 11kb positive sense RNA genome encoding three structural proteins and seven non-structural proteins. Of the three structural proteins, the envelope (E) protein is the major surface protein on the virus and therefore is the primary antigen that elicits the neutralizing antibody response. The mature DENV virion is composed of 180 copies of E proteins. Previous studies showed that DENV2 changes from a smooth compact to a loose bumpy surface morphology when the temperature is raised from 28°C to 37°C [8, 9]. On the smooth compact particles, the E proteins are tightly packed together [10]: three E protein dimers lie parallel to each other forming a raft, and thirty such rafts are organized into a herringbone pattern (S1 Fig). There are extensive interactions between the E proteins in the intra-dimer, inter-dimer and inter-raft interfaces. However, in the bumpy particles, the increased temperature loosens the tight interactions between the E proteins breaking all inter-dimer, inter-raft and some intra-dimer interactions [8, 9] (S1 Fig). This results in the E proteins moving outwards, forming a larger diameter virion particle [8, 9].

The quaternary arrangement of the E proteins on the smooth and bumpy surface particles are therefore very different from each other. It was shown previously that the highly neutralizing antibodies against the smooth particles mainly bind across surface exposed regions of the E proteins at the inter-dimer interface, locking the E proteins together and preventing the structural reorganization necessary for fusion of the virus with the endosomal membrane [11]. However, in the bumpy particles, the inter-dimer relationship between the E proteins is completely broken; these types of antibodies may therefore not be able to bind and neutralize the virus [8, 12]. The individual E proteins on the bumpy surfaced particles exhibit higher solvent accessibility than the smooth particles; as a result, antibodies binding to lower-order E protein assemblies such as the dimer or monomer could then be more effective [12–14]. Conversely, antibodies binding to these lower-order E protein assemblies may not bind to the smooth particles well, as the epitope may be partially or completely hidden due to the tight interactions between the E proteins. This may also potentially lead to lower antibody occupancy, increasing the chance of ADE.

The different morphologies of mature DENV2 therefore impart very different antigenic properties. Despite the morphological differences, both viruses are infectious [8] and thus may pose an additional complication for vaccine development. Here we sought to answer the following questions: (1) Which E protein residue(s) have an impact on conferring the ability of DENV2 to change its morphology at 37°C, and how does this happen at the molecular level? (2) Since the bumpy particles were observed in laboratory adapted DENV2 strains, do they exist in clinical isolates? (3) If the clinical isolate does become bumpy, what temperature is required for this structural transition? Answering these questions should inform on strategies for vaccine and therapeutics development.

Here we identified two DENV2 New Guinea C (NGC) strains with different passage histories from two different laboratories; one is routinely passaged in BHK-21, while the other is passaged in C6/36 cells, showing smooth or bumpy surface morphologies at 37°C, respectively. Comparison of these two strains showed five relatively chemically conserved amino acid changes. A series of E protein mutations were performed on the infectious clone of the smooth DENV2 NGC particle. We showed that subtle mutations at several places can make the virus bumpy at 37°C. Examination of four clinical strains showed three with smooth surface structures at 37°C, and all became bumpy at 40°C. We identified two distinct residues that when individually mutated, switched the virus to bumpy surfaced at 37°C; these residues exist at the intra-dimer interface, and molecular dynamics simulations revealed how they destabilized the E protein intra-dimeric interactions at the atomic level.

## Results

### Identification of residues sensitive to DENV2 NGC morphological changes at 37°C

**Fig 1:**
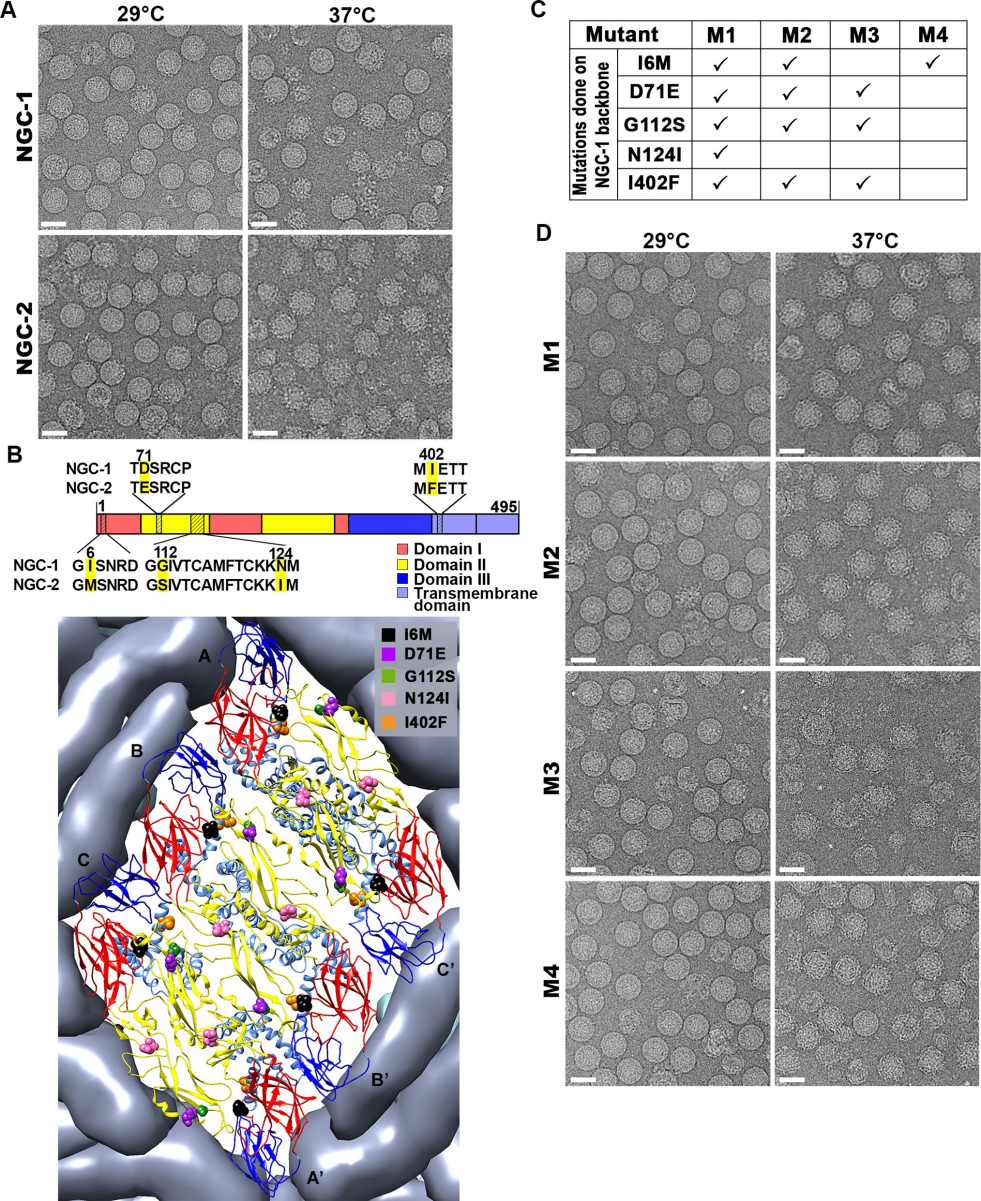
DENV2 NGC strains have different morphologies at 37°C which may be attributed to differences in their E protein sequences. **(A)** CryoEM micrographs of the lab-adapted DENV2 NGC strains with different passage histories (NGC-1 and NGC-2) at 29°C and 37°C. **(B)** (Top panel) Sequence alignment of NGC-1 with NGC-2, and (bottom panel) their residue differences indicated on an E protein raft on the virus surface. (C) List of mutations in M1-4 viruses, and **(D)** the cryoEM micrographs showing their morphologies at 29°C and 37°C. Scale bar is 50nm.

CryoEM micrographs and 2D-averages of the particles of two DENV2 NGC strains from different labs (with different passage histories) (Fig 1A), hereafter named NGC-1 and NGC-2, showed the surface of NGC-1 particles mostly remains smooth with some broken particles at 37°C, while NGC-2 particles turned bumpy (S5A Fig, S6 Fig). Comparison of their E protein sequences showed five differences at amino acid positions 6, 71, 112, 124 and 402 (Fig 1B). These differences do not involve drastic changes in the electrostatic characteristics of the residues. At positions 6, 71 and 402, the differences between NGC-1 and NGC-2 are in the bulkiness of residues while maintaining their charge and polarity: I6M, D71E and I402F. G112S results in an increased in hydrophilicity, whereas conversely, N124I causes an increase in hydrophobicity. In the 3.5Å resolution DENV2 cryoEM structure [15], the side chains of residues 71 and 124 are not involved in E-to-E interactions and are projected towards the outside environment (Fig 1B). Residue 402 is present on the E protein helical stem region and is facing the viral lipid membrane. None of the five residues exist at the E protein inter-dimer or inter-raft interfaces (Fig 1B) in the smooth surfaced DENV2 structure[15]. Only residue 6 lies at the E protein intra-dimer interface.

To identify the determinant(s) that enable the particles to turn bumpy at 37°C, we conducted mutagenesis studies to change all five residues (positions 6, 71, 112, 124 and 402) on the E protein of the smooth surfaced NGC-1 infectious clone to those observed in NGC-2 (Fig 1C). Simultaneous substitution of all five residues (I6M, D71E, G112S, N124I and I402F) in an M1 mutant showed that the particles turned bumpy at 37°C (Fig 1D). Since the M1 mutant contains the E protein sequence identical to NGC-2, but the rest of the structural and non-structural proteins belong to NGC-1, this suggests that its ability to become bumpy is solely due to determinants on the E protein.

M2, M3 and M4 mutants (Fig 1C) were generated and all resulted in more particles becoming bumpy at 37°C (Fig 1D, S5 Fig, S7 Fig). In the M2-4 mutants, residue 124 was not mutated because this residue is facing outwards and not involved in any E-E protein interactions. M2 contains four substitutions (I6M, D71E, G112S and I402F), and showed a bumpy surface (Fig 1D, S5 Fig, S7 Fig) indicating that one or a combination of these mutations is important for causing the change in morphology. M3, which carries three (D71E, G112S and I402F) of the four substitutions in M2, showed a bumpy surface but the particles were more structurally unstable, as more broken particles were observed compared to the other mutants. This suggests that the extra substitution at residue 6 in M2 compared to M3 leads to the stabilization of the bumpy particle. Interestingly the M4 mutant, which contains only one mutation, I6M, also turned the smooth surface virus into bumpy particles.

In conclusion, there are redundancies in substitutions in various positions that turn the virus bumpy, and only subtle changes in the chemical characteristics of the residues are required.

### Characterization of thermal stability and growth curve of the DENV2 NGC mutants

**Fig 2:**
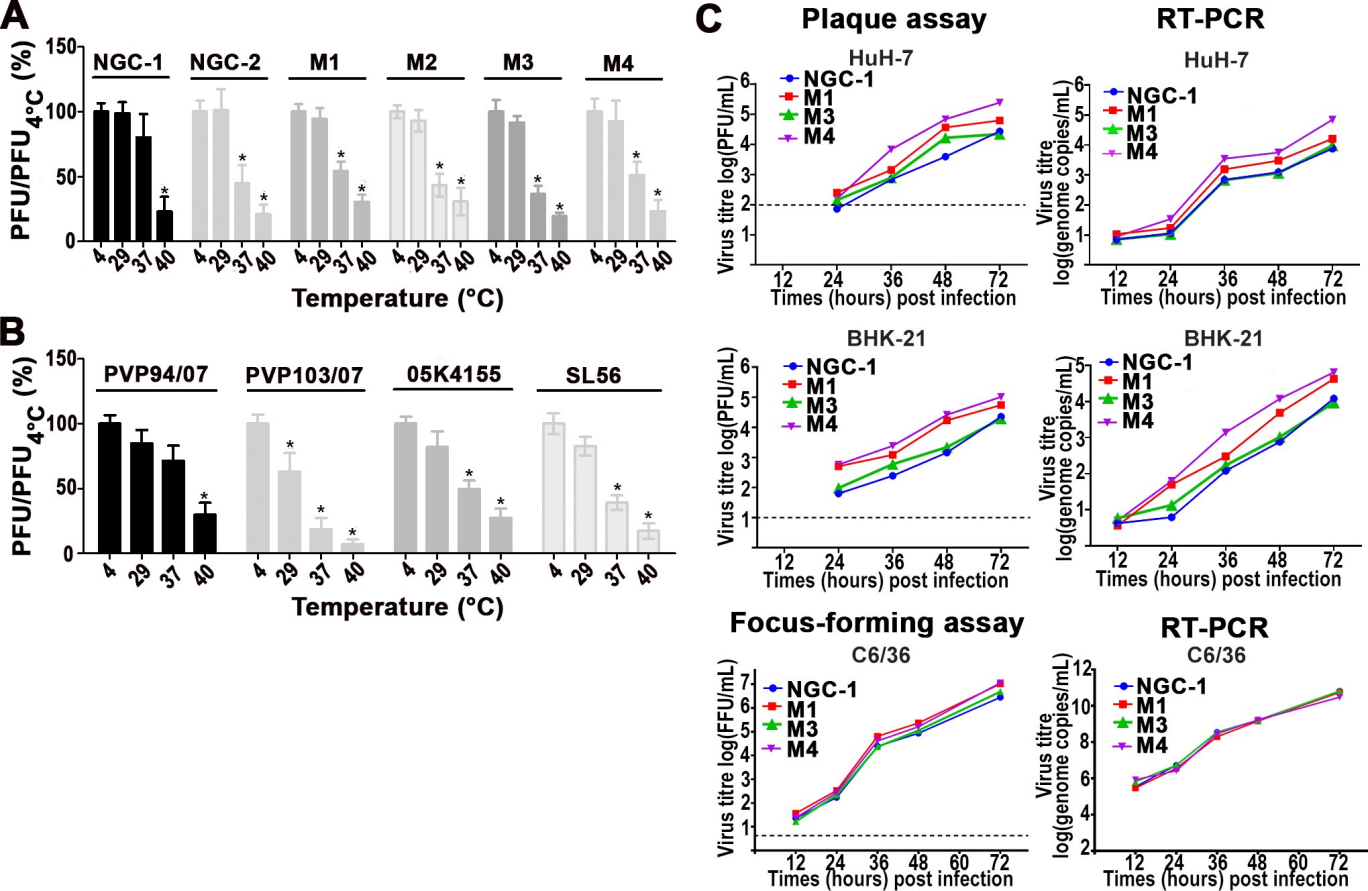
Characterization of the thermal stability and growth curve of the DENV2 strains and mutants. Thermal stability of **(A)** DENV2 NGC strains and mutants, and **(B)** clinical isolates (Table 1) at various temperatures. The y-axis shows the percentage of PFU at different temperatures divided by that of the same virus at 4°C. *P<0.01 (paired t-test for A, B). **(C)** Growth curve of DENV2 NGC-1, M1, M3 and M4 in both mammalian (HuH-7, BHK-21) and mosquito cells (C6/36) detected by plaque assays (HuH-7, BHK-21), focus-forming assay (C6/36) and RT-PCR (HuH-7, BHK-21 and C6/36). Dashed line represents the limit of detection of the assay. All assays have been repeated at least 3 times.

To test the thermal stability of the DENV2 NGC mutants, we pre-incubated the viruses at different temperatures (4°C, 29°C, 37°C and 40°C) for 30 mins before conducting plaque assay at 29°C using BHK-21 cells. These temperatures were chosen to mimic the physiological temperature in the mosquitoes (29°C), human host (37°C) and also when humans are experiencing high fever (40°C). The percentage of plaques, for each incubation temperature of virus, were calculated by comparing to the same virus pre-incubated at 4°C. For the DENV2 NGC strains (NGC-1 and NGC-2) and mutants (M1-4) (Fig 2A), all showed similarly high stability when pre-incubated at 4°C and 29°C. At 40°C, they were all equally unstable. The most obvious differences in stability between NGC-1 compared to the other viruses occurred at 37°C, with the NGC-1 strain significantly more stable than the others. This is correlated with their differences in morphologies between NGC-1 (smooth) and the other viruses (bumpy) at 37°C, while at 29°C and below, they have smooth surfaces.

Separate growth curve experiments were performed (Fig 2C), where NGC-1, M1, M3 and M4 mutants (without pre-incubation at respective temperatures) were allowed to infect mammalian cells (HuH-7, BHK-21) at 37°C, and mosquito cells (C6/36) at 29°C. All viruses were infected at the same multiplicity of infection (MOI) of 0.1. The growth curves were followed by taking samples at different time points and the amount of virus in the supernatant determined by either plaque or focus-forming assays, and real-time polymerase chain reaction (RT-PCR). 12 hours post infection of virus in HuH-7 and BHK-21 cells (37°C) showed similar quantities of virus by RT-PCR, suggesting that they bind to the cells with equal efficiencies. However, starting at 24 hours post-infection, both plaque and RT-PCR started to show differences in growth rate between the NGC mutants, largely with the smooth surfaced NGC-1 growing slightly slower than the bumpy viruses (M1 and M4). However, the smooth surfaced NGC-1 virus and the bumpy surfaced M3 virus growth curves largely overlap in the mammalian cell, which may be due to the structural instability of the M3 mutant as observed by cryoEM (Fig 1D). When tested in C6/36 cells where virus is grown at 29°C, all viruses have similar growth curves; this is expected as they all have smooth surfaces at this temperature. Together, the results suggest that morphological differences between mutants at 37°C likely play an important role in affecting their growth.

Interestingly, the NGC-1 and M4 strains in mammalian cell lines (HuH-7 and BHK-21) showed the biggest differences in their growth rate, yet they differ by only one residue (I6M). Not considering M3, as the particles were structurally unstable, the bumpy particles formed by the M1 and M4 mutants seemed to have slightly higher growth rates than that of the smooth surface NGC-1 strain in mammalian cells.

In conclusion, the smooth surface particles (NGC-1) seemed to be more thermally stable than the bumpy particles (NGC-2 and M1-4). In terms of growth rate in mammalian cells, the bumpy particles (M1 and M4) showed slightly faster growth rates than the smooth particle (NGC-1) while in C6/36 cells where all viruses are grown at 29°C, they would have similar smooth surface morphologies and thus could lead to the observed similar growth rate.

### Morphology of the DENV2 clinical isolates at 29°C, 37°C and 40°C

**Fig 3:**
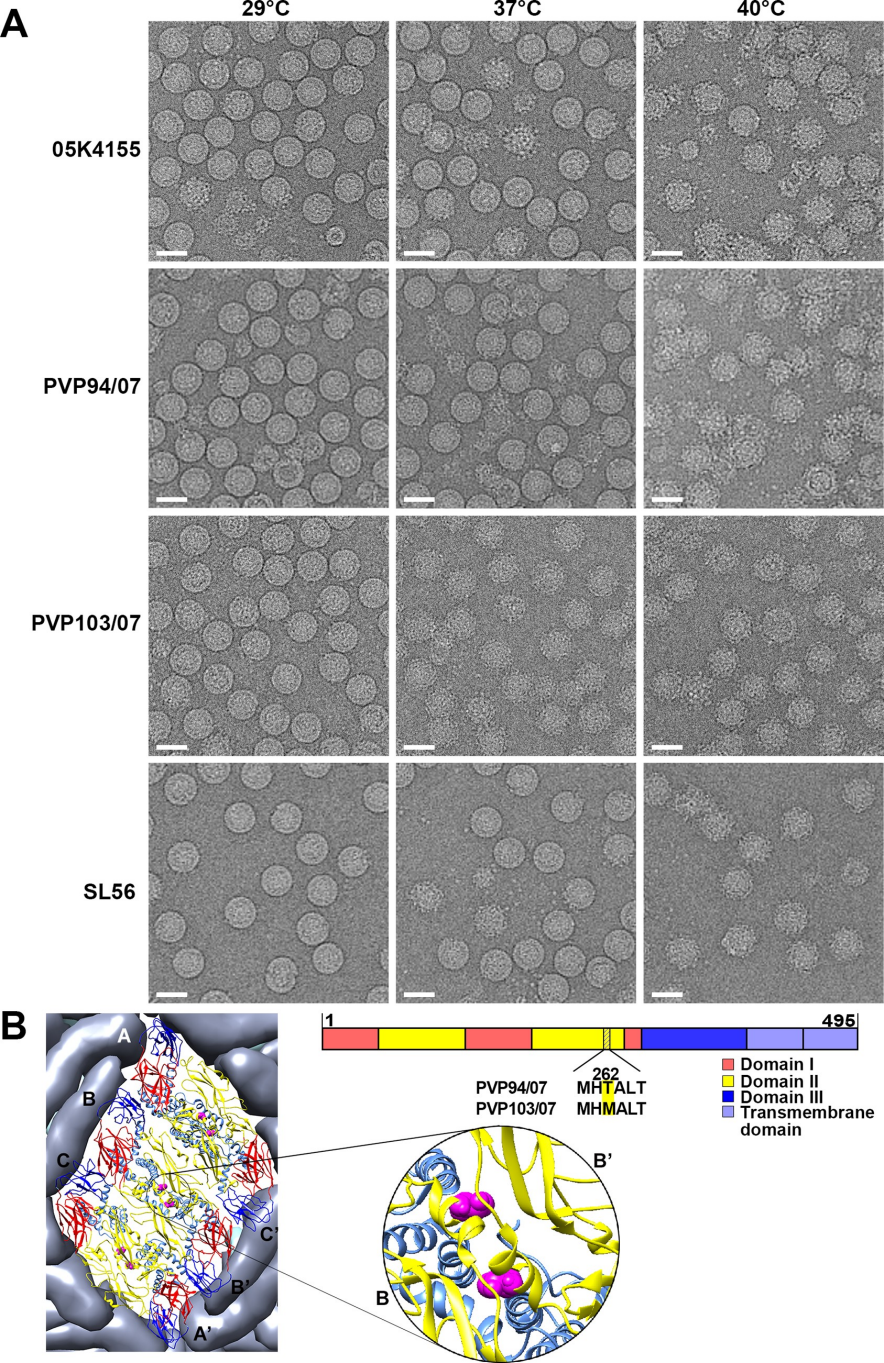
Morphology of DENV2 clinical strains at different temperatures. **(A)** CryoEM micrographs of DENV2 clinical isolates (PVP94/07, PVP103/07, 05K4155 and SL56) at pH7.4 at 4°C, 37°C and 40°C. Scale bar is 50nm. **(B)** (Right) Sequence alignment of PVP94/07 and PVP103/07 showing only one residue difference at position 262 of E protein, and (left) its location on an E protein raft on the virus surface.

Previous studies showing DENV2 particles [8, 9] that can become bumpy at 37°C were mostly observed in laboratory adapted strains: 16681[9], NGC [8], and WRAIR strain S16803[8]. We tested the naturally circulated DENV2 strains for their morphological changes at both 37°C and 40°C. Four clinical strains (05K4155, PVP94/07, PVP103/07, SL56) were used, three were isolated from Singapore and one from Sri Lanka. PVP94/07 and PVP103/07 were both isolated in the same year (2007) in Singapore suggesting they are from the same outbreak, whereas the rest are from different outbreaks (Table 1). The viruses were grown in C6/36 cells; the part of the viral genome encoding prM-E of the particles used for cryoEM were sequenced and showed that limited passages (~4–7 passages) did not result in additional mutations compared to the original viruses. Particles of DENV2 PVP94/07, 05K4155 and SL56 strains exhibited a smooth surface morphology at 37°C whereas PVP103/07 appeared bumpy (Figs 3A, S5B and S8). This suggests that the clinical strains can adopt both morphologies. When heated to 40ºC mimicking high fever conditions, all the clinical strains became bumpy (Figs 3A, S5B and S8).

**Table 1:** Strains of DENV2 clinical isolates used in this study.

Virus strain	Year of isolation	Country of origin
05K4155	2005	Singapore
PVP94/07	2007	Singapore
PVP103/07	2007	Singapore
SL56	2009	Sri Lanka

Thermal stability tests of the clinical strains at 37°C were done (Fig 2B), and the strains were ranked from the most to the least stable in the following order: the smooth surfaced strains PVP94/07, 05K4155, SL56, and finally the bumpy surfaced PVP103/07. This shows a similar trend as that observed with the NGC mutants, suggesting that the bumpy particles of clinical strains at 37°C likely cause them to be less stable than the smooth particles.

Interestingly, DENV2 PVP94/07 and PVP103/07 which were isolated within the same year are different in morphology: PVP94/07 (smooth) and PVP103/07 (bumpy), yet their E protein sequences differ by only one amino acid, at position 262: threonine in PVP94/07 and methionine in PVP103/07 (B). Therefore, one could be a mutant of the other.

The T262M substitution between PVP97/07 and PVP103/07 is located at the intra-dimer E protein interface (Fig 3B). Interestingly, the mutation of a single residue, I6M, in the NGC-1 E protein (Fig 1C), which is also located at the intra-dimer interface, also changed the smooth surfaced virus to bumpy. This suggests that destabilization of the E protein dimer may play an important role in controlling the morphology of particles at 37°C.

### Comparison of the characteristics of the smooth surfaced (PVP94/07) to bumpy surfaced (PVP103/07) DENV2 clinical strains

**Fig 4:**
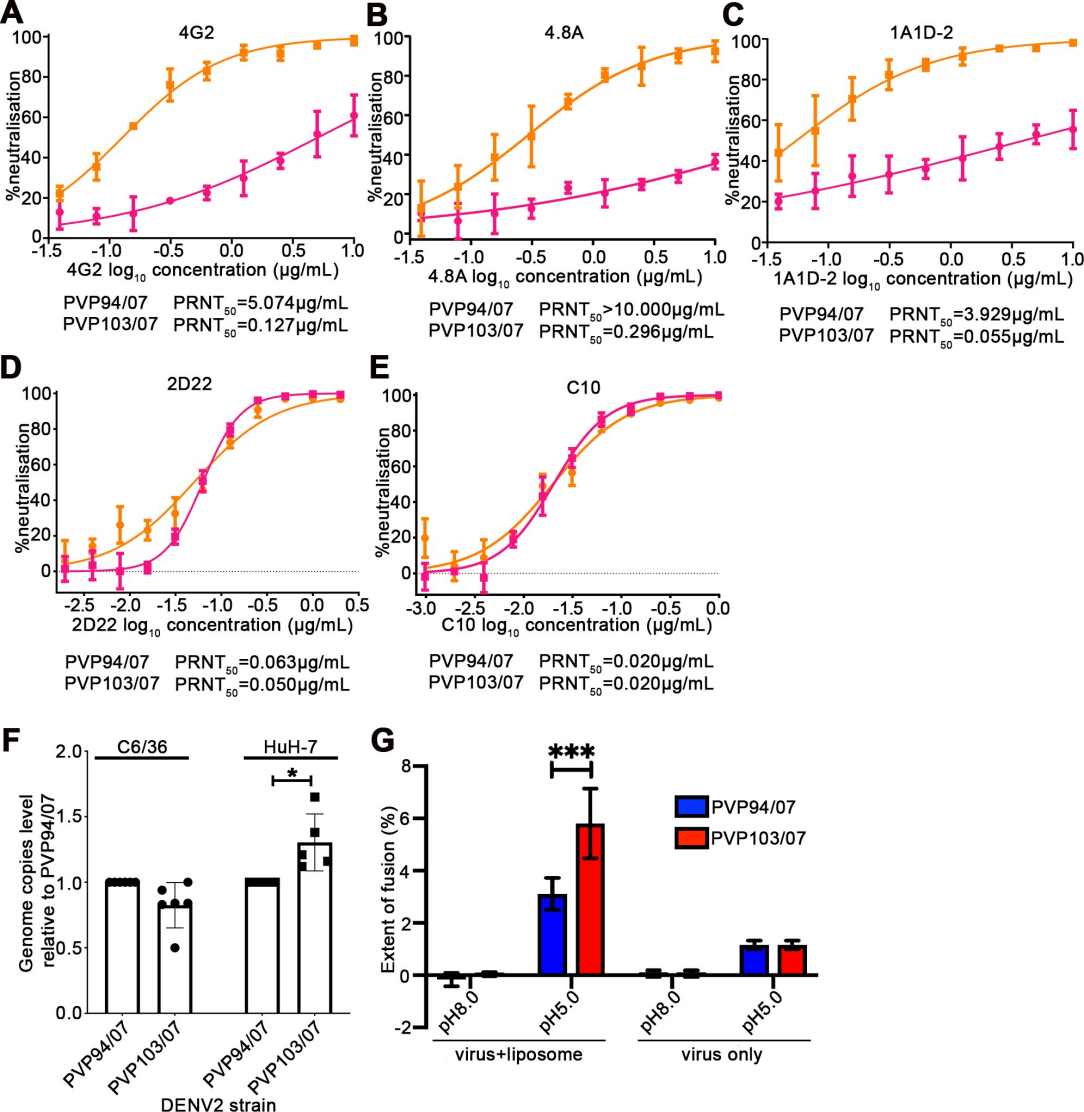
Characterization of the smooth PVP94/07 and bumpy surfaced PVP103/07 DENV2 clinical isolates. **(A-E)** Neutralization profiles of MAbs **(A)** 4G2, (B) 4.8A, **(C)** 1A1D-2 **(D)** 2D22 and **(E)** C10 against PVP94/07 and PVP103/07. Data points represent average of n = 3 experiments with SD error bar. **(F)** Comparison of the attachment ability of PVP103/07 and PVP94/07 to C6/36 and HuH-7 cells. Y-axis shows genome copies level relative to PVP94/07, details of the formula is in the methods section. More bumpy surface particles (PVP103/07) are observed to bind to HuH-7 than the smooth surfaced PVP94/07 while no significant difference is detected when these virus are exposed to C6/36. Each data point represents one independent experiment with error bar representing SD. (* p<0.05) **(G)** Fusion assay of PVP94/07 and PVP103/07 with liposomes showed that bumpy surfaced PVP103/07, has a better fusion efficiency to liposomes. The average with standard error calculated from averaging the replicates of three individual experiments each with triplicates is presented. A two-way ANOVA followed with Fisher’s LSD test was carried out to compare the extent of fusion (%) of DENV2 PVP94/07 with DENV2 PVP103/07 at pH 5.0. ***p = 0.0010 shows the fusion efficiency is significantly different between PVP94/07 and PVP103/07.

There is only one amino acid difference in the E protein, T262M, between PVP94/07 (smooth) and PVP103/07 (bumpy) strains. This allows us to study the difference in the neutralization profile of various antibodies to viruses that have different morphologies but minimal amino acid sequence variation in their E protein. We used antibodies that have been shown to be neutralizing and bind to different regions on the E protein. Monoclonal antibodies (MAb) 4G2 [16, 17] and 4.8A [18] were shown to bind to E protein domain II. The epitope for 4G2 is thought to be partially hidden on the smooth virus surface [16]. Epitopes bound by MAbs 1A1D-2 [19], 2D22 [12] and C10 [20] had been previously shown by crystallography or cryoEM. MAb 1A1D-2 bind to domain III and the epitope is partially exposed on the compact smooth virus surface [19] while the MAb 2D22 and C10 recognize epitopes across the E protein dimer (EDE antibodies). MAb C10 when exposed to soluble E proteins can assemble the monomeric E proteins into dimers [20]. We tested their neutralization profiles to DENV2 PVP94/07 (smooth) and PVP103/07 (bumpy) strains. MAbs 4G2, 4.8A and 1A1D-2 have differing neutralizing capabilities to the different virus morphologies. They neutralized the bumpy surface virus better than the smooth surfaced one (Fig 4A–4C), suggesting that their epitopes are more accessible in the bumpy surface morphology. The EDE antibodies 2D22 and C10, on the other hand, neutralized both morphologies equally well (Fig 4D and 4E).

We characterized the efficiencies of the smooth (PVP94/07) and bumpy (PVP103/07) surfaced DENV2 to attach to cells, and also to fuse with liposomes—steps that mimic the early cell entry process. To determine the cell attachment capabilities of the smooth (PVP94/07) and bumpy (PVP103/07) surfaced DENV2 (virus structural changes were induced by incubation at 37°C), we determined the amount of these viruses bound to 4°C pre-cooled *Aedes albopictus* C6/36 and human HuH-7 cells by quantitative RT-PCR. Results showed there were slightly less bumpy PVP103/07 particles bound to C6/36 cells compared to the smooth PVP94/07 DENV2, however, their difference is not statistically significant (Fig 4F). As for HuH-7 cells, we detected significantly larger amounts of bound PVP103/07 compared to PVP94/07 (Fig 4F). We also characterized the ability of the smooth (PVP94/07) and bumpy (PVP103/07) surfaced DENV2 to fuse with liposomes at pH 5.0. We detected higher levels of fusion of PVP103/07 to liposomes than PVP94/07 (Fig 4G). Together the results suggest that the bumpy surfaced virus is better than the smooth surfaced virus particles in attaching to cells and fusing with the endosomal membrane during cell entry. These observations are consistent with the higher growth rate of the bumpy NGC M4 mutant, which has a substitution at amino acid position 6 of the E protein (also at the E protein dimer interface), than the smooth surfaced NGC-1 in HuH-7 cells (Fig 2C).

### Molecular dynamics simulations showing individual substitutions, T262M and I6M, on E proteins affecting their intra-dimer interactions

**Fig 5:**
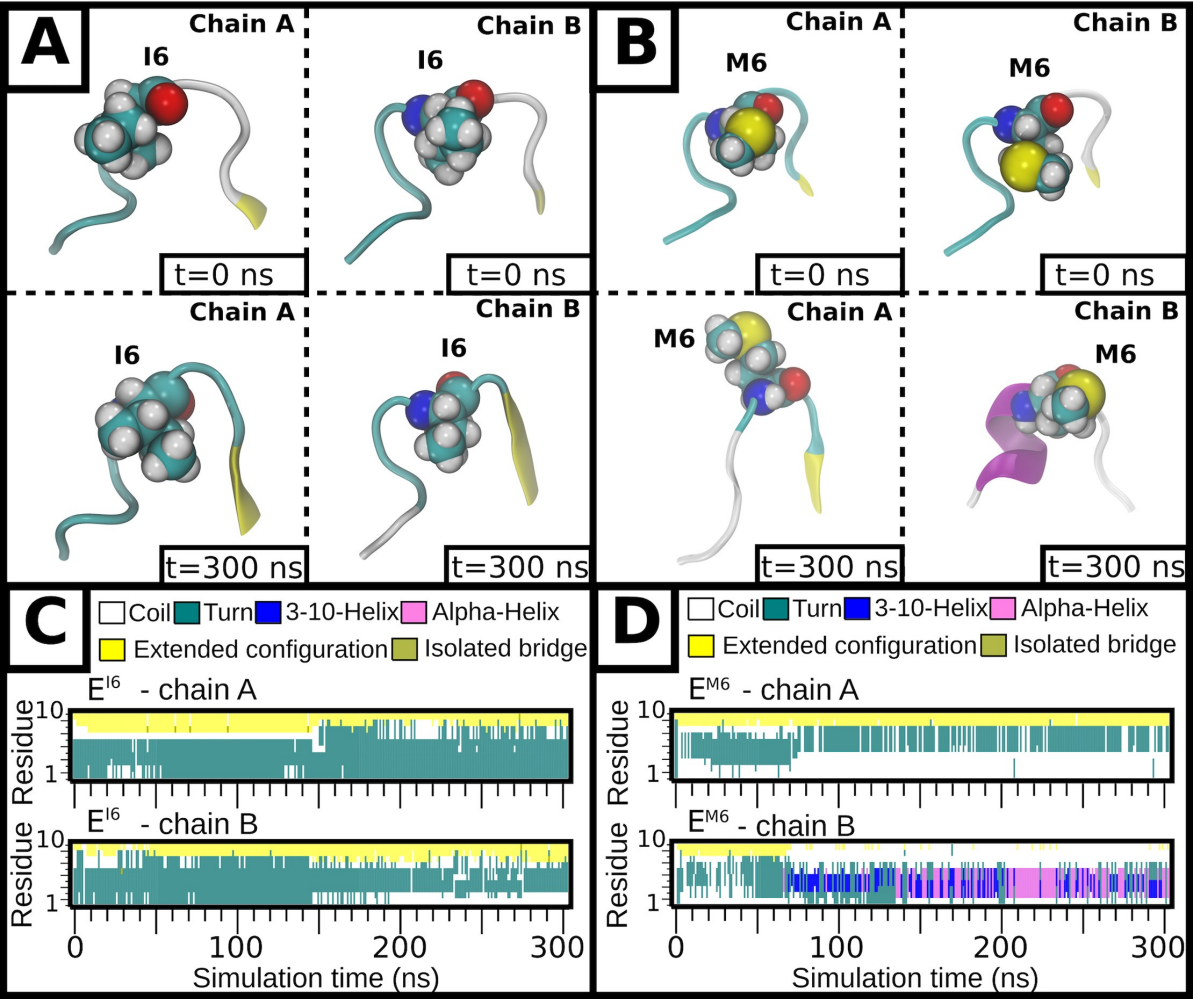
MD simulations showing the stability of the N-terminal loop of the E protein protomers within a dimer in the E^I6^ and E^M6^ systems. The initial (t = 0 ns, top) and final (t = 300 ns, bottom) conformations of the N-terminal loop (residues 1–10) are shown for both protomers (chains A and B) of the **(A)** E^I6^ and **(B)** E^M6^ systems. The protein backbone is shown as ribbons and colored according to the secondary structure. Residue number 6 is shown as sphere (carbon in cyan, nitrogen in blue, oxygen in red and hydrogen in grey). The per-residue secondary structure propensity for the N-terminal loop over simulation time is shown for each chain in the **(C)** E^I6^ and **(D)** E^M6^ systems.

We have shown that the single point mutation I6M in the DENV2 NGC-1 E protein changes the surface of the virus to bumpy at 37°C (Fig 1). In order to explore the structural basis for this change, we have employed atomically detailed, explicitly solvated molecular dynamics (MD) simulations of the NGC-1 (E^I6^) and M4 (E^M6^) states using the ~800 residue E protein ectodomain dimers for E^I6^ and E^M6^ at 37°C (see details in Methods). The N-terminal loop encompassing residues 1–10 retained its cryoEM determined dimeric conformation in E^I6^ over the 300 ns simulation trajectory (S2 Fig). The I6 side chain of both E protein protomers within a dimer remained embedded within the interior of this well-defined turn throughout the simulations (Fig 5A and 5C). In contrast, a conformational change was observed in each N-terminal loop of the E^M6^ protomers within the dimer within <100 ns (S2 Fig). This was due to the presence of the bulkier sulfur atom of the methionine thioether group, which led to its sidechain flipping outwards (Fig 5B), influencing the local secondary structure of the loop (Fig 5B and 5D). In chain A, the M6 substitution led to an extended loop structure, while that in chain B led to the formation of a helical structure (Fig 5B and 5D). The latter is in agreement with the propensity for methionine to favour α-helical conformations [21]. This “local switch” in the I6M mutant also caused spontaneous global changes across the E protein dimer, highlighted by filtering the “noise” from the trajectory using principal component analysis (PCA) in order to visualize the largest-amplitude motions, as shown in S1 Movie. Domain I was observed to shift “outwards” with respect to domain III of each chain (Fig 5), and domain II also then reoriented leading to a change in the relative position of the two antiparallel helical segments at the centre of the dimeric complex. This would be expected to disrupt the symmetry-related contacts on the smooth virus surface, leading to it becoming bumpy at 37°C.

**Fig 6:**
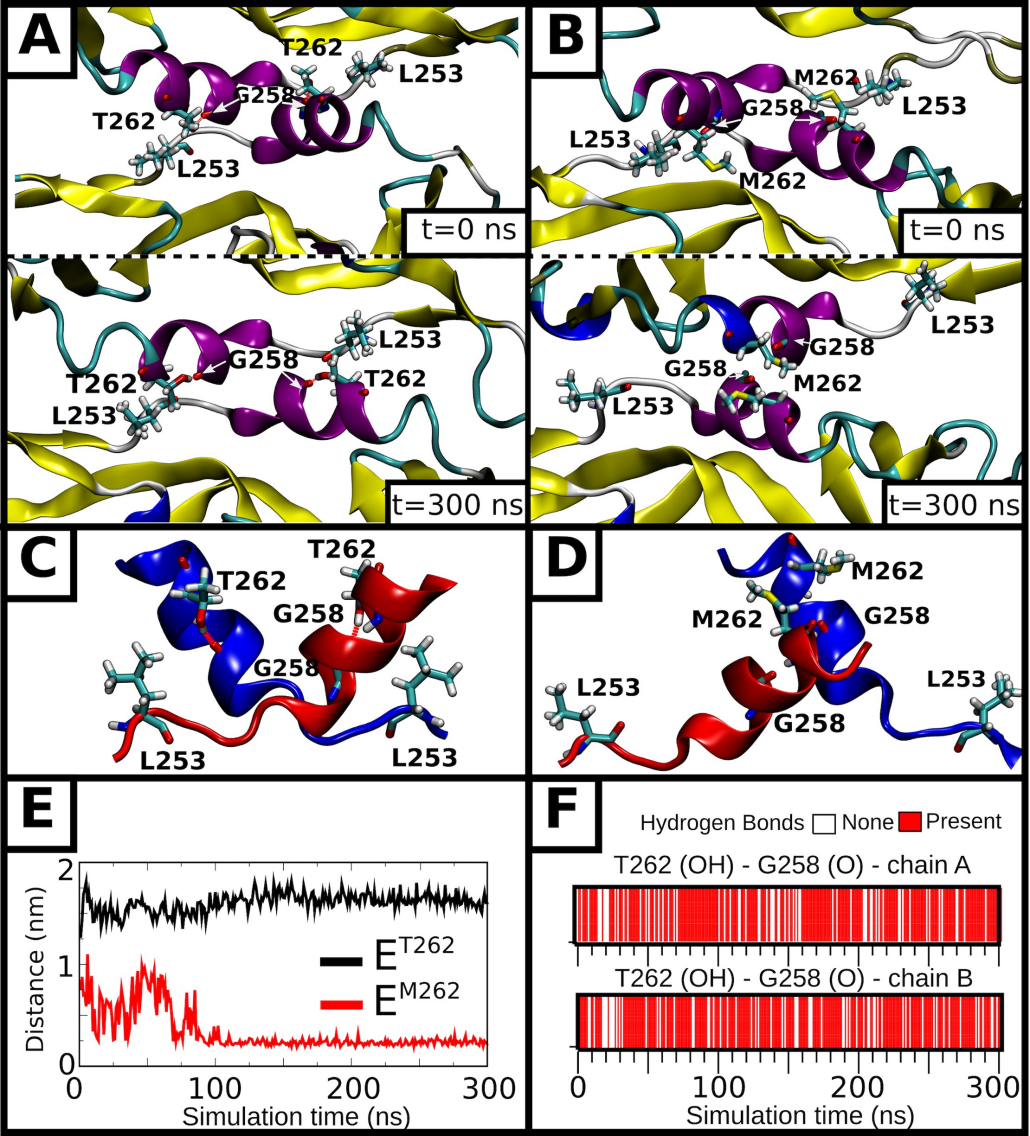
MD simulations showing stability of the central helical interface of the E proteins within a dimer in both E^T262^ and E^M262^ systems. The initial (t = 0 ns, top) and final (t = 300 ns, bottom) states, visualized along the dimer axis, are shown for the **(A**) E^T262^ and **(B)** E^M262^ systems. Key residues side chains are shown as stick representation (carbon in cyan, nitrogen in blue, oxygen in red and hydrogen in grey) and are accordingly labeled. The remainder of the protein is shown as ribbon and colored according to the secondary structure (3_10_-helix in blue, α-helix in purple, extended configuration in yellow, loop in cyan, coil in white). A side-on view of this region (residues 250–265 of both chains) is shown for the final states of **(C)** E^T262^ and **(D)** E^M262^, with either chain colored in blue or red. In **(E)**, the minimum distance over simulation time between residue number 262 from opposing chains is shown for the two systems. In **(F)**, existence maps for the intra-helical hydrogen bond between the T262 side chain hydroxyl and the G258 backbone carbonyl oxygen are shown for each E protein chain in the E^T262^ system.

Comparison of DENV2 clinical isolate PVP94/07 with PVP103/07 showed the T262M substitution in the E protein resulted in the virus becoming “bumpy” at 37°C (Fig 3). We employed a similar MD simulation protocol as above to study the dimer stability of both E^T262^ and E^M262^ systems. The E^T262^ showed a stable conformation relative to the cryoEM structure over 300 ns (S2 Movie). The relative orientation of the two E protein chains was well maintained (Fig 6A), while the two antiparallel helical segments encompassing residues 250–265 at the centre of the dimeric complex retained their experimentally defined interface in terms of buried surface (Fig 8A) and structure (Fig 6A and 6C) throughout the simulation time, with the T262 side chains of opposing chains separated by ~1.6 nm (Fig 6A, 6C and 6E). A persistent intra-helical hydrogen bond between the T262 side chain hydroxyl and the G258 backbone carbonyl contributed to the stability of this helical region (Fig 6F). In contrast, E^M262^ was significantly less stable compared to the E^T262^ (S3 Movie). The loss of the intra-helical hydrogen bond between M262 and G258 led to the rearrangement into a new interface that was stable over hundreds of nanoseconds (Fig 6B and 6D). This was accompanied by the formation of a hydrophobic interaction between the side chains of the two M262 residues each from an opposing chain, with a final separation of ~0.25 nm (Fig 6E), as indicated by the total buried area between the central helical segments (Fig 8A). This resulted in a large-scale conformational change in the E protein dimer, as illustrated via PCA (Fig 8B and S4 Movie). Thus, in both protomers, domain II shifted outwards with respect to domains I and III, while domain III in only one of the protomers (Fig 6B) was observed to turn towards domain II of the other protomer (Fig 6B). These changes are expected to disrupt the symmetry-related contacts in the smooth compact virus structure, leading to the virus turning bumpy as observed in our cryoEM micrograph of the respective clinical isolates (Fig 3).

## Discussion

Changes in DENV2 morphology will influence its recognition by antibodies. Questions on whether this morphological diversity occurs in those circulating in nature, and if primary E protein sequence analysis alone can accurately predict the morphology, are important for vaccine and therapeutics design. To answer the question on whether primary sequence can inform on the morphology of DENV2, we mutated different individual or sets of multiple residues of the E protein of the bumpy surface DENV NGC (NGC-2) into the infectious clone of the smooth surfaced NGC (NGC-1) virus. The results showed that most mutations—even chemically subtle ones—changed smooth viruses into bumpy surfaced ones, suggesting multiple regions of the virus E protein could destabilize the surface. Since subtle mutations at different positions can have an effect on the DENV2 morphology, it is therefore unlikely that one could predict with confidence, from the primary sequence alone, the morphology of DENV2. Goo et al., 2017 [22], showed yet another mutation, T198F (from small polar side chain (T) to bulky hydrophobic side chain (F)) on the DI-DII hinge, not included in our study, that makes a previously cryptic epitope on DIII more accessible on both WNV and DENV1 for antibody binding. The authors suggested that due to the mutation, the virus likely becomes bumpy, thereby increasing the accessibility of the epitope on DIII for antibody neutralization. However, they did not show the morphology of the virus with cryoEM.

**Fig 7:**
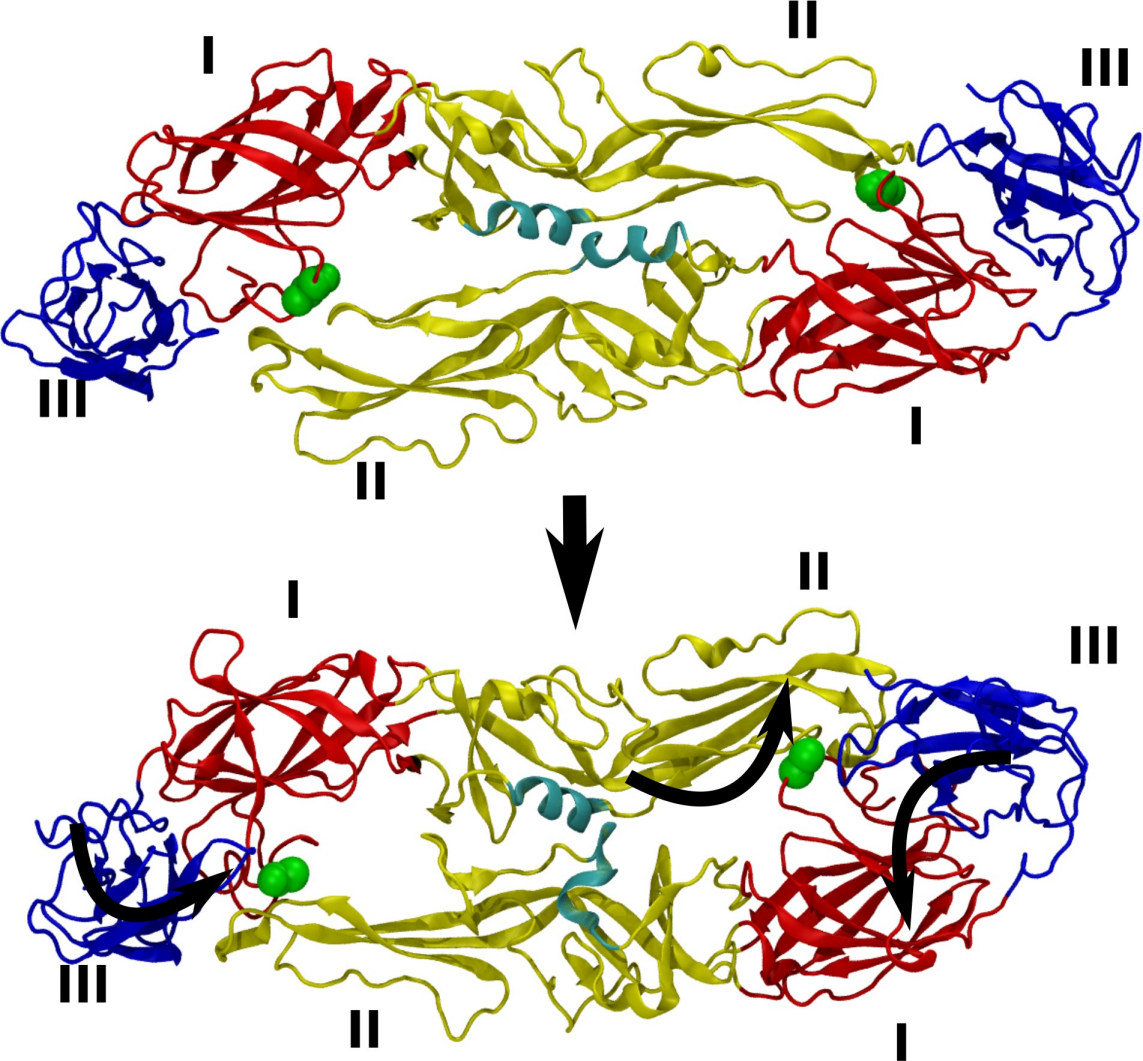
MD simulations showing conformational changes in the E protein dimer induced by E^I6^ to E^M6^ mutation. The two extreme conformations generated by principal component analysis (PCA) from a filtered trajectory representing the dominant motion of the E protein dimer in E^M6^ are shown. The protein is shown as ribbons, with red, yellow, and blue corresponding to domains I, II, and III, respectively. The helical interface (residues 250–265) between the two chains of the dimer is shown in cyan. M6 backbone atoms are shown as green spheres. Curly arrows represent the directions of motion of domains I, II, and III.

**Fig 8:**
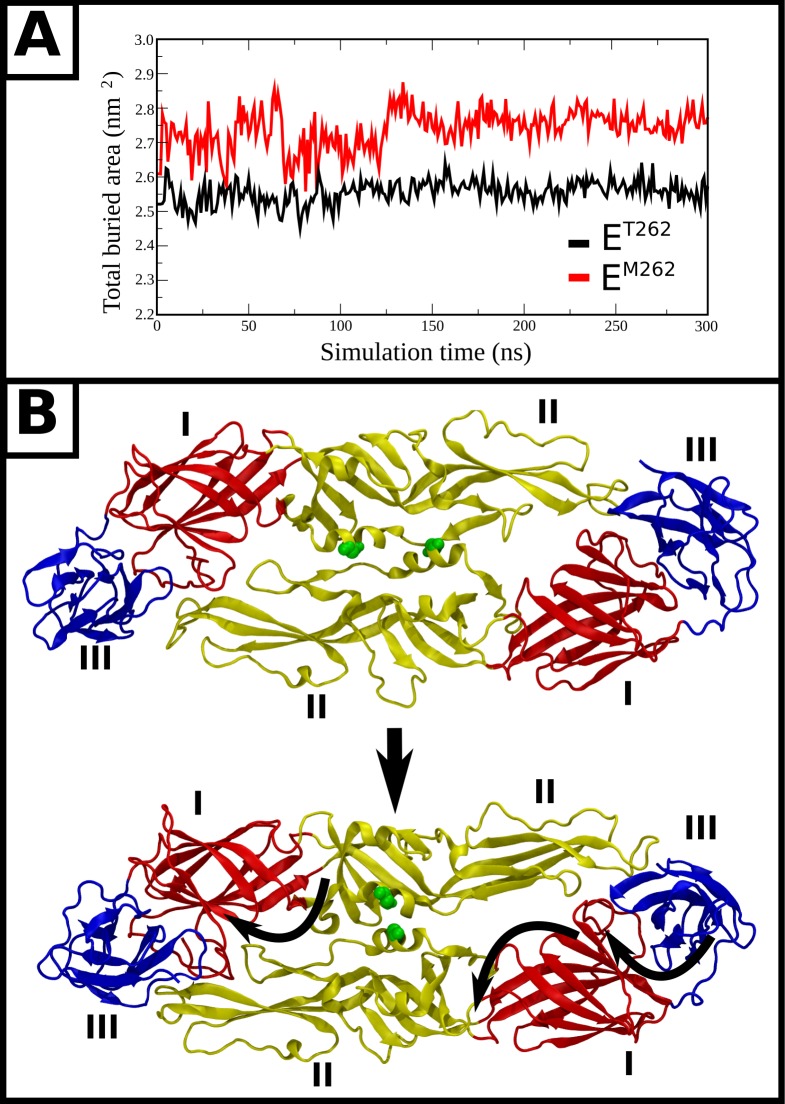
MD simulations showing conformational changes in the E protein dimer induced by mutation of E^T262^ to E^M262^. **(A)** The total buried surface area between helical residues 250–265 from opposing chains is shown with respect to simulation time for both systems. **(B)** The two extreme conformations generated by principal component analysis (PCA) from a filtered trajectory representing the dominant motion of the E protein dimer in E^M262^ The protein is shown as ribbons, with red, yellow and blue corresponding to domains I, II, and III respectively. M262 backbone atoms are shown as green spheres. Curly arrows represent the directions of motion of domains I, II, and III.

The I6M mutation that changes the smooth surface NGC strain into the bumpy form (M4 mutant) (Fig 1), and the T262M difference between clinical isolates (PVP94/07 and PVP103/07) (Fig 3) that results in PVP103/07 having bumpy surface at 37°C, are located at the interface of the E-E protein dimeric interactions. MD simulations showed how these substitutions (Fig 7 and Fig 8) destabilized the E protein dimer and they may then cause larger scale changes in the quaternary arrangements of the E proteins on the smooth virus particle surface.

In this study, we were able to observe one laboratory-passaged DENV2 NGC strain from an infectious clone, having a smooth surface morphology. In contrast, other laboratory-adapted strains (WRAIR strain S16803, 16681 and our lab DENV2 NGC) were shown to have a bumpy surface morphology at 37°C. As for the clinical isolates examined, three out of four strains have smooth surface at 37°C. This may suggest that there could be a selective pressure *in vivo* for smooth surfaced particles. Thermal stability tests of the NGC mutants showed the smooth surfaced virus (NGC-1) is the most thermally stable virus compared to the bumpy surfaced mutants at 37°C (Fig 2A). Previous studies comparing dengue viruses to Zika virus, which has a smooth surface even at 40°C, showed Zika virus retains infectivity after incubation at 40°C while the DENV viruses are comparatively much lower in infectivity at elevated temperatures. This correlation suggests the smooth viral surface imparts thermal stability [23]. However, other than the difference in their surface morphologies, these viruses also have significant sequence differences in other parts of the genome, thereby adding more variability in the interpretation of the data. The latter possibility could be excluded by a previous study using chimeric Zika and DENV, showing that when the viral structural prM-E genes from these viruses were switched, this also switches their thermostability characteristics [24].

Although the smooth NGC-1 particles are more thermally stable (Fig 2A), the growth curve (without pre-incubating virus at respective temperatures) (Fig 2C) in mammalian cells showed them to have a slightly slower growth rate than the bumpy (M1 and M4) particles at 37°C. This correlates with our attachment and fusion assay results comparing the smooth PVP94/07 and bumpy PVP103/07; the PVP94/07 particles were shown to have a lower extent of attachment to human cells and also reduced fusion to liposomes (Fig 4F and 4G). We speculate that perhaps in the host, the cells for DENV are not readily available, and the viruses may need to travel a distance to find their next target cell and therefore, their thermal stability at 37°C in the absence of host cells becomes a more important factor than the growth rate in infected cells, leading to selection of the smooth particles. In the lab, we generally passage DENV in C6/36 cells at 29°C, and at this temperature, all viruses (regardless of whether they carry substitutions that lead to a bumpy surface at 37°C) will have smooth surfaces, and therefore the selection pressure is low. We have shown previously that the DENV2 NGC strain (NGC-2), when incubated at 37°C, contains four structural classes of particles—one class is the smooth surface particles, while the other four classes are expanded particles that looked bumpy. Since our thermal stability tests showed that the smooth surface particles are more stable, it could be possible that one can select for smooth particles by pre-incubating the NGC-2 at 37°C for 30 mins before infecting BHK-21 cells at 37°C; however, this may require multiple passages. Co-incidentally, DENV2 NGC-1 which has a smooth surface, has been continuously passaged in BHK-21 cells at 37°C.

Our studies showed that the *in vitro* versus the *in vivo* passaged virus may display largely different morphologies and comparison of their E protein sequences showed some residue differences (S3 Fig). Although they have largely chemically conserved residues, our studies showed even subtle changes can cause major morphological changes. This suggests that the use of lab adapted strains may not be completely representative of the viruses naturally circulating when studying antigenicity. This was also shown in a study by Chaichana et al. [25] in which it was demonstrated that laboratory-adapted DENV2 strain 16681 displayed higher levels of ADE compared to patient-derived DENV2. Leah et. al. [26] using human sera neutralization cartography have also shown that strains within a serotype can be as antigenically diverse as between DENV serotypes. We therefore suggest that laboratory experiments should use seed virus cultures derived from infectious clones of the clinical isolates displaying different morphologies, so as to ensure translatability of the results to viruses in nature. We detected that limited passaging (4–7 passages) in C6/36 cells of the clinical isolates did not cause any E protein mutations in the virus, however, extended passaging should best be avoided.

The capability of antibodies to neutralize viruses may depend on the accessibility of the respective epitope(s), which will vary depending on the virus surface morphology. For example, the E protein fusion loop is only partially exposed on the smooth surface DENV and therefore, antibodies targeting this region can only achieve partial occupancy which may lead to ADE [27, 28], whereas on the bumpy particles, this region is much more accessible, allowing antibody neutralization. Antibodies that are highly neutralizing against the smooth surfaced DENV1 [13, 29], DENV3 [11] and WNV [30] particles have been shown to bind across E protein dimers, a conformation highly dependent on the preservation of the quaternary structure of the smooth surface virus. These antibodies would not be able to bind to the bumpy particles as the E proteins are arranged differently. Indeed, comparison of the neutralization activities of several antibodies (that bind to different regions of the E proteins) to the smooth PVP94/07 and bumpy surfaced PVP103/07 DENV2 clinical isolates, (Fig 4A–4E) showed that they can have different potencies against different virus morphologies. We showed here that for the four clinical strains, three have a smooth surface while one is bumpy at 37°C; this suggests that for vaccine development, DENV2 should include both morphologies so that the vaccine may then elicit protective antibody responses to all morphologies. As for antibody therapy, for prophylactic use where the patient body temperature is at 37°C, an antibody mixture that binds efficiently to both smooth and bumpy particles should be used. For therapeutics, where usually the patient is experiencing fever, our cryoEM data shows that all clinical strains at 40°C appear to have bumpy surfaces, suggesting that antibodies that bind efficiently to lower-order assemblies of E proteins (monomers or dimers) should be used.

## Materials and methods

### Cells and viruses

*Aedes albopictus* clone C6/36 (American Type Culture Collection (ATCC) CRL1660, USA) and BHK-21 (ATCC CCL10) cells were cultured in RPMI 1640 medium supplemented with heat-inactivated 10% fetal bovine serum (FBS) (Gibco, Brazil) and incubated in 5% CO_2_ at 29°C and 37°C respectively. HuH-7 (Japanese Collection of Research Bioresources (JCRB) Cell Bank JCRB0403, Japan) cells were cultured in DMEM medium supplemented with heat-inactivated 10% FBS and incubated in 5% CO_2_. DENV2 clinical isolates were from the Early Dengue infection and outcome study (EDEN) (05K4155, PVP94/07 and PVP103/07; Singapore)[31] and Aravinda de Silva (SL56; Sri Lanka).

### Construction of mutant DENV cDNA clone

The general procedure for construction of DENV cDNA has been described elsewhere. [32]. Briefly, a single E protein mutation was first introduced into a shuttle vector using a QuikChange III XL site-directed mutagenesis kit (Stratagene). The mutated DNA fragments from the shuttle vectors were sequenced and then swapped into the pACYC-FL-NGC cDNA clone through. For triple or pentadruple mutations (D71E-G112S-I402F or I6M-D71E-G112S-N124I-I402F), multiple rounds of mutagenesis were performed to engineer all mutations into a shutter vector. Each round of mutagenesis introduced one mutation into the shutter vector and the introduced mutation was confirmed by sequencing. The fragments containing the mutations were then engineered into the pACYC-FL-NGC plasmid by using restriction enzymes BsrGI and NheI. All cDNA plasmids were again verified by DNA sequencing.

### In vitro transcription, RNA transfection, and immunofluorescence assay (IFA)

The assay protocols used were described previously [32]. Briefly, RNAs were transcribed from linearized full-length cDNA plasmids using T7 mMessage mMachine Transcription kit (Thermo Fisher Scientific) and then electroporated into BHK-21 cells. Viral E protein synthesis in transfected cells was monitored by IFA with DEN-immune mouse 4G2 (American Type Culture Collection) and anti-mouse immunoglobulin G conjugated with fluorescein isothiocyanate (FITC) as the primary and secondary antibodies, respectively. The culture fluid was harvested on day 4 or 5 post transfection and stored at -80°C for subsequent plaque assays.

### Virus sample preparation

The method for virus production and purification has been described previously [34]. The clinical isolates used in this study were passaged not more than 7 times. Briefly, all the strains of DENV2 were propagated in *Aedes albopictus* C6/36 cells at 29°C at 5% CO_2_. The infection was carried out at a confluency of 80% and at MOI of 0.1 for 2h. This was followed by the replacement of the inoculum with fresh media containing 2% FBS. The viruses were harvested 4 days post-infection and centrifuged to remove cell debris. Precipitation of the virus from the media by 8% w/v polyethylene glycol 8000 in NTE (10mM Tris-HCl pH8, 120mM NaCl and 1mM EDTA) was carried out overnight at 4°C followed by centrifugation through a 24% w/v sucrose cushion. This was further purified using a 10 to 30% w/v potassium tartrate gradient. The virus band was then extracted, concentrated through a 100k-Da filter and buffer exchanged to NTE buffer. The virus purity was verified using Coomassie Blue-stained SDS-PAGE gel to ensure suitability for cryoEM imaging.

### CryoEM sample preparation

The clinical isolate strains were adjusted to pH7.4 using 1M Tris-Cl pH7.4 at a final concentration of 100mM Tris-HCl. The various strains of DENV2 were aliquoted and incubated at their respective temperature (4°C, 37°C or 40°C) for 30 min followed by ~2h at 4°C. Subsequently, 2.2 μL of the virus sample was pipetted to a carbon-coated lacey carbon grid (Ted Pella), blotted with filter paper, snap frozen in liquid ethane using FEI Vitrobot Mark IV and stored in liquid nitrogen.

### CryoEM image acquisition

The virus particles were imaged using a Titan Krios cryo-electron microscope equipped with a field emission gun of 300kV. The images were detected using a direct electron detector (Falcon, FEI) and collected manually. Images were collected at 47,000x magnification with pixel size of 1.71 Å. Contrast transfer function parameters were estimated using the Gctf programme[35]. For each image dataset, particle picking was done manually using the e2boxer in EMAN2 [36] and then 2D classification was done on these particles using Relion2.1[37].

### Viral genome sample preparation for sequencing and sequence analysis

The preparation of viral genome for sequencing was modified from Christenbury JW *et al* (2010). Briefly, viral RNA was extracted from purified virus using QIAamp Viral RNA Mini Kit in accordance to manufacturer’s protocol. cDNA was synthesized from the viral RNA using SuperScript III First-Strand Synthesis System according to manufacturer’s protocol with primer D2a5B [38]. Subsequently, the cDNA was amplified in a polymerase chain reaction (PCR) with high fidelity PfuUltra II Fusion HS DNA polymerase using previously reported primers d2s1C and d2a18 [38]. The PCR products were then run in a 1% agarose gel containing 1X SYBR Safe and visualized. Upon determining the absence of non-specific products, the PCR products were purified using Qiagen MiniElute PCR Purification Kit. Purified PCR products were then quantified using Nanodrop. The purified PCR products were submitted for single pass DNA sequencing using primers listed in S1 Table that covers overlapping regions. Primers used in DNA sequencing were designed from the multiple sequence alignment of DENV2 sequences available in NCBI Nucleotide database. Chromatograms obtained from DNA sequencing were analysed in FinchTV software. The sense sequences were verified against the antisense sequences. The regions that encoded for prM and E protein were extracted and translated using ExPASy translate tool. Sequence alignment was carried out on the translated sequences using Clustal Omega.

### Plaque assay to determine stability at different temperature

Each virus strain was diluted to about 500 PFU/ml and split to four aliquots, followed by incubation at 4°C, 29°C, 37°C and 40°C respectively for 30 min. Subsequently, each virus strain at the different temperatures were used to infect BHK-21 seeded in 24-well plates for 1.5 hour at 29°C before washed with phosphate-buffered saline (PBS), overlaid with carboxy-methyl cellulose and incubated at 37°C. After 3–5 days, the cells were fixed and stained. Plaque forming units (PFU) were then tabulated as percentages to that of the control (4°C).

### Plaque reduction neutralization assay (PRNT)

The neutralization activities of MAb 4G2, 4.8A, 1A1D2, 2D22 and C10 to clinical isolates PVP94/07 and PVP103/07 were determined by PRNT. Both DENV2 strains PVP94/07 and PVP103/07 were pre-incubated at 37°C for 30 min to induce structural changes before they were exposed to MAbs. Two-fold serial dilutions of each MAb were done and then they were incubated with equal volumes of virus at 37°C for 30 min. 100 μL of these mixtures were layered on BHK-21 cells in 24-well plates and incubated at 37°C for 1 h. The infected cells were washed with PBS, overlaid with carboxyl-methyl cellulose and then further incubated at 37°C for 3–4 days. Cells were fixed and stained. Percentage neutralization was determined from the comparison of the number of plaque forming units (PFU) in each antibody dilution to the control without MAbs. PRNT_50_ is the concentration of the antibody that results in 50% reduction in PFU.

### Virus growth kinetics

HuH-7 (Japanese Collection of Research Bioresources (JCRB) Cell Bank JCRB0403, Japan), BHK-21 and C6/36 cells were seeded into 12-well plates. The next day, the cells were infected with DENV WT and mutants at MOI of 0.1. The MOI determined using plaque assay in BHK-21 was used for the assays on HuH-7 and BHK-21 cells. The MOI determined using focal forming assay (FFA) in C6/36 was used for the assays on C6/36 cells. After 1 h incubation, the virus inocula were removed. The cells were washed twice with phosphate buffered saline and cultured with 1 ml of fresh medium per well. The culture medium was collected at indicated time points and stored at -80°C. Virus titers were determined by using RT-PCR and either plaque assays (samples from HuH-7 and BHK-21 cells) or FFA (samples from C6/36 cells).

### Focal forming assay

C6/36 was seeded in 24 well plate and incubated overnight. Virus samples were 10-fold serially diluted before infecting the cells for 1h at 29°C. The cells were then overlaid with carboxy-methyl cellulose and incubated at 29°C for 4 to 5 days. Thereafter, the overlay was removed and cells were washed with phosphate buffered saline before being fixed with 80% acetone. The cells were then blocked with 5% milk (Anlene, New Zealand) then incubated with 4G2 followed by horseradish peroxidase conjugated anti-mouse antibody. The foci were developed using 3,3'-diaminobenzidine substrate (Dako). The focus forming unit (FFU) were counted and tabulated.

### Attachment assay

DENV2 strains PVP94/07 and PVP103/07 were pre-incubated at 37°C for 30 min to induce structural changes. C6/36 and HuH-7 grown in 24-well plate were pre-cooled at 4°C before incubated with the DENV2 strains PVP94/07 and PVP103/07 at multiplicity of genome containing particles (MOG) of 10,000 at 4°C for 1 hour. After incubation, the cells were washed 3 times with cold PBS and RNA were extracted using RNeasy Mini Kit (Qiagen). cDNA was synthesized using qScript cDNA supermix. Taqman real-time PCR was performed using a mixture of primers specific to virus and also housekeeping β-actin gene; listed in S2 Table [39–42]. The genome copies of the DENV2 strains were normalized to the housekeeping gene β-actin and the relative fold difference of the genome copies of the strain PVP103/07 to that of PVP94/07 was determined using the following 2^-ΔΔCT^ method [43]:

$$Relative\;change\;in\;copy\;number=2^{-[(C_{T,PVP103/07}-C_{T,\beta -actin})-(C_{T,PVP94/07}-C_{T,\beta -actin})]}$$

### Virus:Liposome fusion assay using DiD-labelled virus

Membranes of the purified DENV2 strains PVP94/07 and PVP103/07 were labelled with the lipophilic fluorescent probe 1,1′-dioctadecyl-3,3,3′,3′-tetramethylindodicarbocyanine, 4-chlorobenzenesulfonate (DiD) dye (Invitrogen) as described previously[44]. Briefly, virus was incubated at 28°C for 30 min with 1 mM DiD dye dissolved in DMSO. The DiD-labelled virus was filtered through a PD-10 desalting column (GE Healthcare) to remove unbound dye. Labelled virus was used within 2 days.

Equal volumes of virus and liposomes were mixed on ice and diluted with 20 μl of 2% BSA in NTE. 60 μl of pre-chilled low pH buffer (50 mM MES) was then added. The total volume of the sample is 100 μl. The fluorescence was recorded at excitation and emission wavelengths of 633 nm and 665 nm, respectively, using a Tecan Infinite M200 microplate reader every minute over 30 minutes at 37°C. The maximum amount of lipid mixing was determined by adding 20 μl of 2% of Triton X-100 and measuring the fluorescence. The concentration of virus used in the experiment was standardized based on the maximum amount of lipid mixing prior to the experiment. The extent of fusion reported is calculated by dividing the experimental fluorescence (at t = 30 mins) with the fluorescence after the addition of Triton X-100 and normalized to the ratio of the extent of fusion between the two viruses alone at pH 5.0.

### MD simulations

The initial conformation used for simulations of the dimeric E protein ectodomain were obtained from the cryoEM structure of DENV2 in its mature state (protein data bank entry: 3J27 [15], residues 1–395) under conditions of neutral pH. For consistency with the experimentally studied strain PVP94/07 (corresponding to the E^T262^ simulation system), several *in silico* point mutations (E47K, Q52H, I61V, D71A, K126E, H149N, I164V, N390S) were introduced using PyMol (https://www.pymol.org). In addition, for strain PVP103/07, the additional T262M mutation was introduced (corresponding to the E^M262^ simulation system). Similarly, for strain NGC-1 (corresponding to the E^I6^ simulation system), the mutation E47K was introduced, along with the additional I6M mutation for strain NGC-M4 (corresponding to the E^M6^ simulation system). Each construct was placed in the centre of a cubic box (20x20x20 nm^3^) and solvated with approximately 120,000 TIP3P[45] water molecules. All simulations were performed using the Amber99SB*-ILDN-Q forcefield.[46] Ionizable residues and termini were treated in their fully charged state, with sodium ions added to neutralize the overall system charge. MD simulations were performed with GROMACS 5.0.2.[47] Equations of motion were integrated with the Verlet leapfrog algorithm using a 2 fs time step. Bond lengths were constrained with the LINCS algorithm.[48] The cutoff distance was switched from 0.9 to 1.2 nm for the short-range neighbour list and van der Waal’s interactions. The Particle Mesh Ewald (PME)[49] method was applied for long-range electrostatic interactions with a 1.2 nm real space cutoff. The velocity rescale thermostat with an additional stochastic term[50] and Parinello-Rahman[51] barostat were used to maintain the temperature at 37°C and pressure at 1 bar, respectively. Initial velocities were set according to a Maxwell distribution. Periodic boundaries were applied in all directions. Initial configurations were minimized using the steepest descent algorithm, followed by equilibration with position restraints on protein heavy atoms with a force constant of 1,000 kJ mol^-1^ nm^-2^ in the *NVT* and subsequently *NPT* ensembles, for 5 ns and 10 ns, respectively. Production runs for each system were generated for 300 ns in the *NPT* ensemble, without restraints. Simulations were performed on an in-house Linux cluster of 7 nodes comprised of 2 GPUs (Nvidia K20) and 20 CPUs (Intel Xeon CPU E5-2680 v2 @ 2.8 GHz) each or the National Supercomputing Centre Singapore (http://www.nscc.sg) using 4 nodes comprised of 1 GPU (Nvidia K40t) and 24 CPUs (Intel Xeon CPU-E5-2690 v3 @ 2.60 GHz) each. Secondary structure was analysed using VMD software.[52] Covariance analysis and PCA[53] was performed for all Cα atoms in each 300 ns trajectory. The first principal component accounted for ~50% of the total variance in each case. Hydrogen bonds were measured based on a donor–acceptor distance cut-off of 0.35 nm and a hydrogen–donor–acceptor angle cut-off of 30°. All structural analyses were performed using tools within the GROMACS and VMD packages.

### Statistical, sequence and structural analysis

Data were analysed and graphs were generated using Graphpad Prism 6 software. Paired student t-test was used to determine significance. Sequence alignment was done using Multialn[54] and depicted using ESPript[33]. Structural analysis was carried out using USCF Chimera [55] on cryoEM structures of compact (PDB accession code 3J27) and expanded DENV2 (PDB accession code 3ZKO).

## Supporting information

S1 Fig Organization of E dimers in the raft of a compact and expanded DENV particle. (TIF)

S2 Fig MD simulations showing the conformational drift. (TIF)

S3 Fig E protein variation between different DENV2 strains. (TIF)

S4 Fig Pairwise alignment of the envelope sequence of PVP94/07 and PVP103/07. (TIF)

S5 Fig Distribution of the different particle morphologies (smooth, rough surfaced and broken particles) within different DENV2 strains at different temperatures. (TIF)

S6 Fig 2D class averages of particles in the micrographs of NGC-1 and NGC-2 at 29°C and 37°C. (TIF)

S7 Fig 2D class averages of the particles in the micrographs of M1, M2, M3 and M4 at 29°C and 37°C. (TIF)

S8 Fig 2D class averaging of the particles in the micrographs of clinical strains 05K4155, PVP94/07, PVP103/07 and SL56 at 29°C, 37°C and 40°C. (TIF)

S1 Movie MD simulations showing oscillation between extreme states obtained via principal component analysis (PCA) from the filtered trajectory representing the dominant motions of the E^M6^ system. (MPG)

S2 Movie MD simulations showing the 300 ns trajectory of the E^T262^ WT system. (MPG)

S3 Movie MD simulations showing the 300 ns trajectory of the E^M262^ mutant system. (MPG)

S4 Movie MD simulations showing oscillation between extreme states obtained via principal component analysis (PCA) from the filtered trajectory representing the dominant motions of the E^M262^ system. (MPG)

S1 Table Primers for sequencing prM-E encoded region of the DENV2 genome. (DOCX)

S2 Table Primers and probes for qRT-PCR. (DOCX)
