# Supplementary figures and images for: Molecular basis of dengue virus serotype 2 morphological switch from 29°C to 37°C
Source: PLoS Pathog. 2019 Sep 19;15(9):e1007996. doi: 10.1371/journal.ppat.1007996 (PMC6752767; doi:10.1371/journal.ppat.1007996)

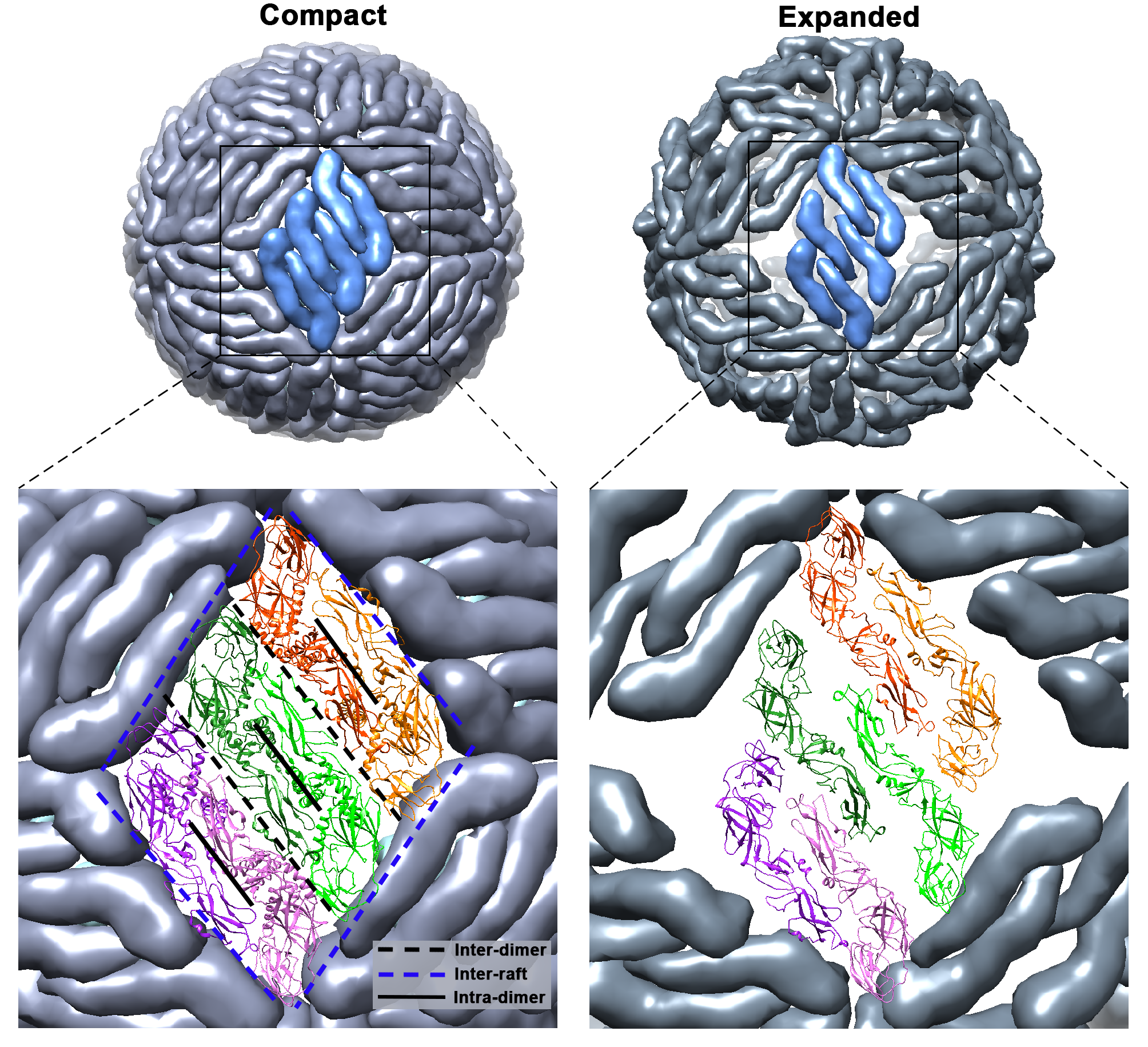

Supplement: S1 Fig — (TIF) [file ppat.1007996.s001.tif]

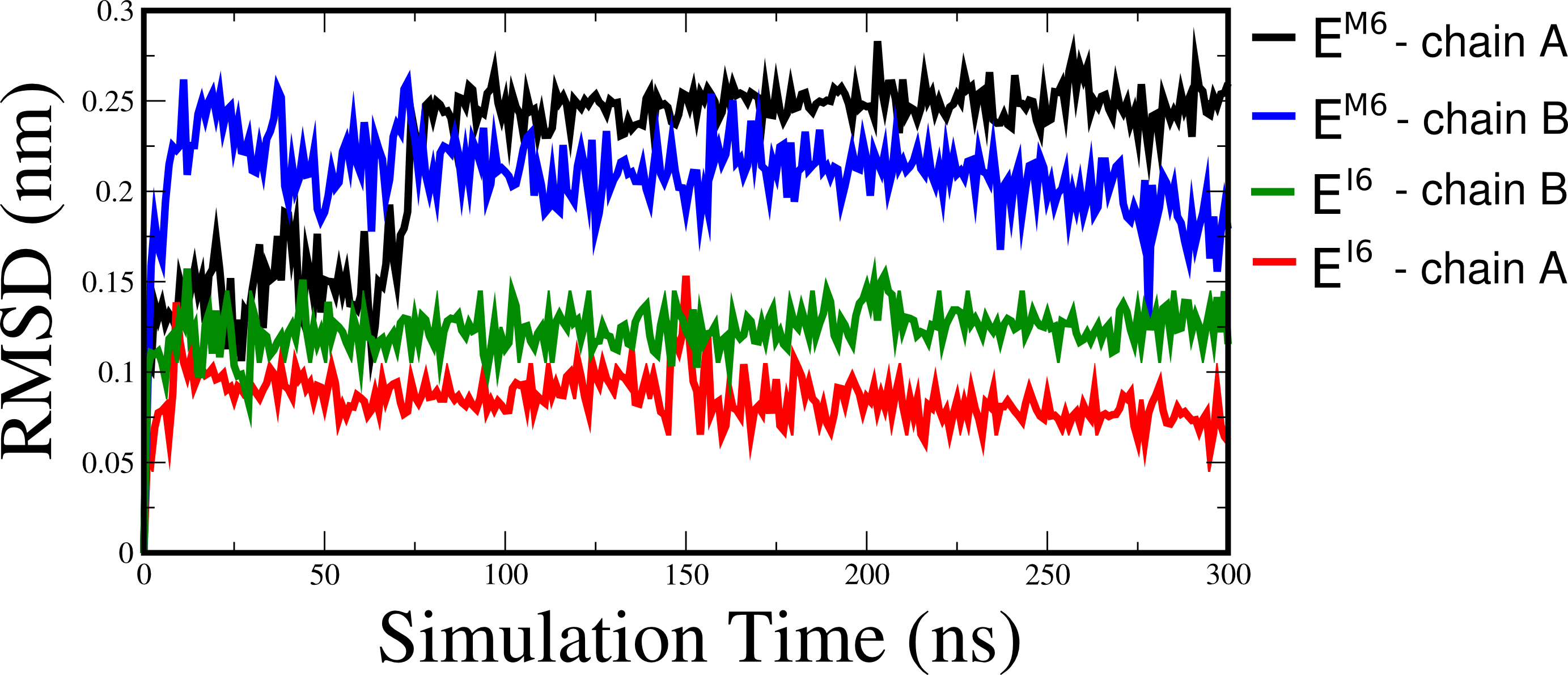

Supplement: S2 Fig — (TIF) [file ppat.1007996.s002.tif]

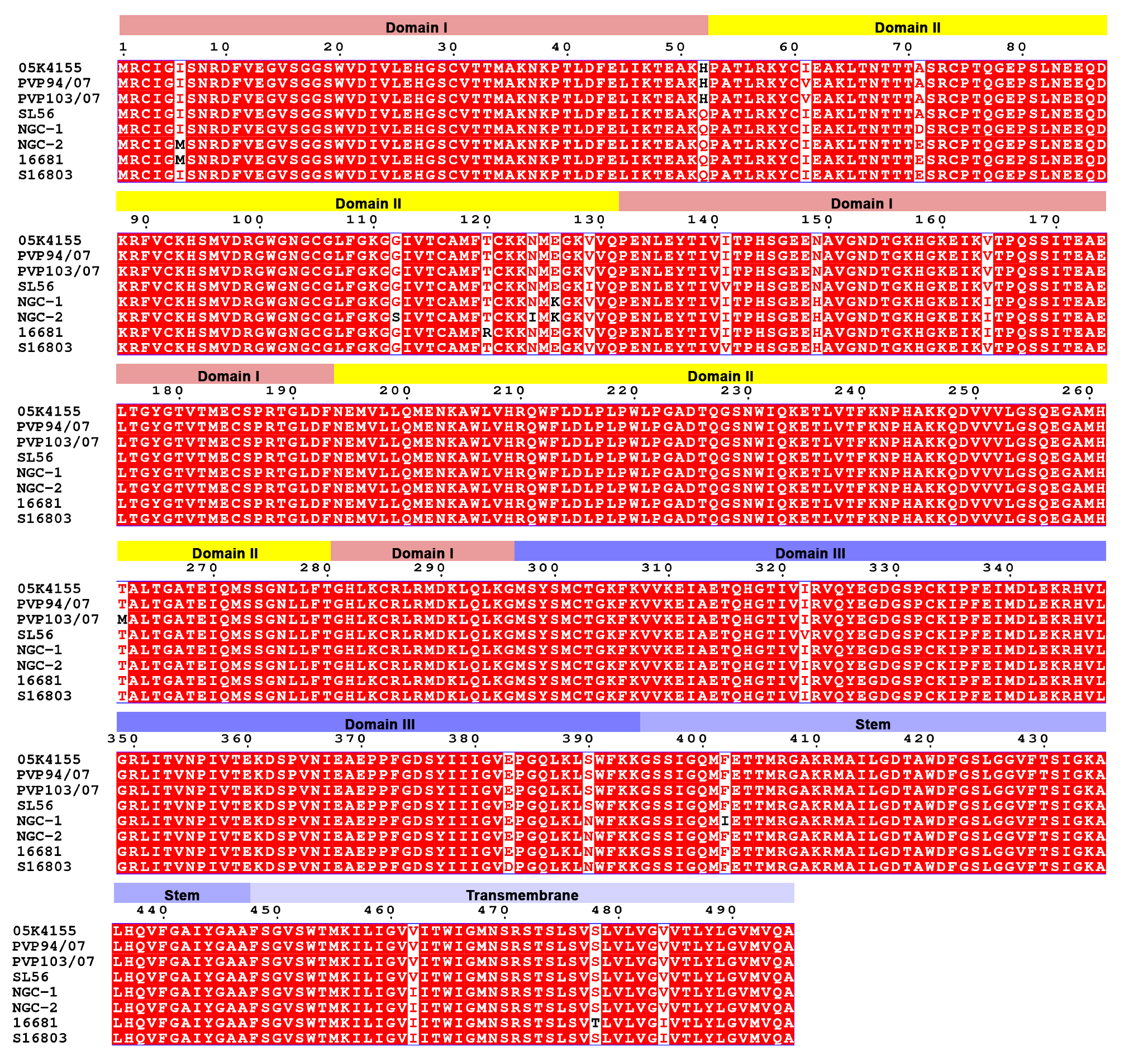

Supplement: S3 Fig — (TIF) [file ppat.1007996.s003.tif]

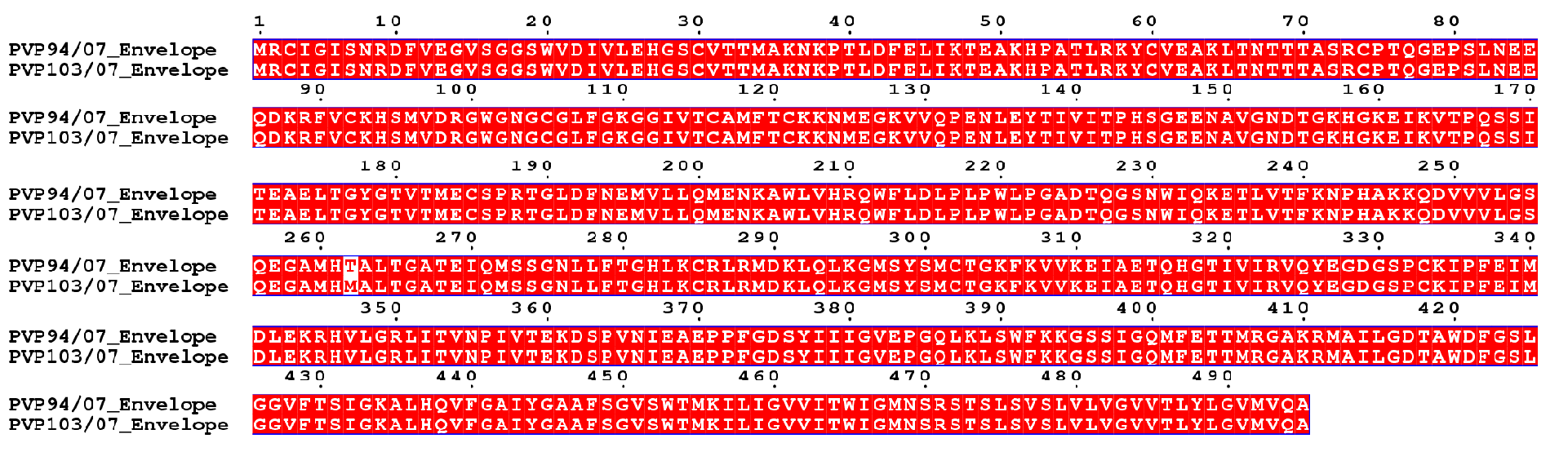

Supplement: S4 Fig — (TIF) [file ppat.1007996.s004.tif]

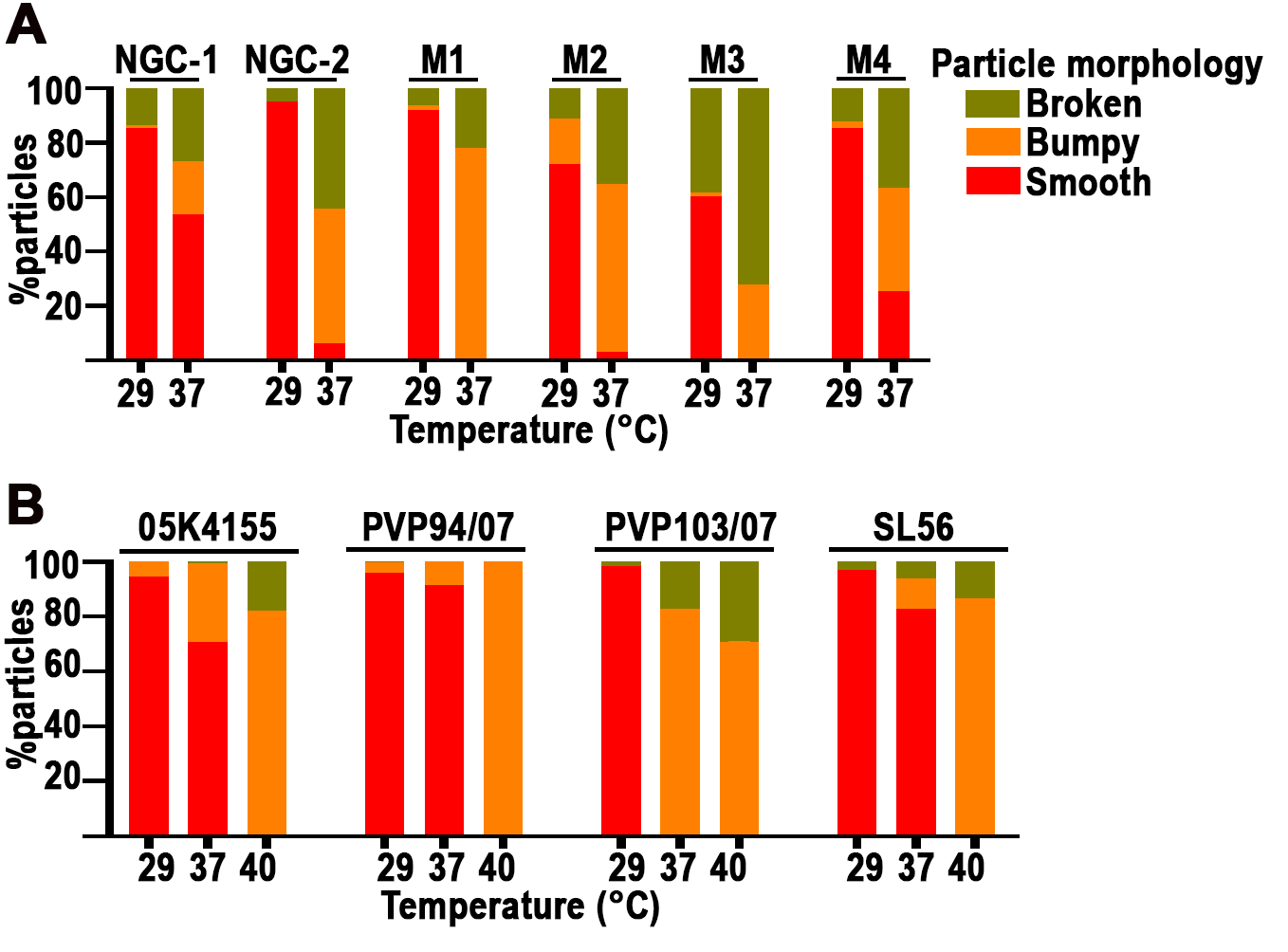

Supplement: S5 Fig — (TIF) [file ppat.1007996.s005.tif]

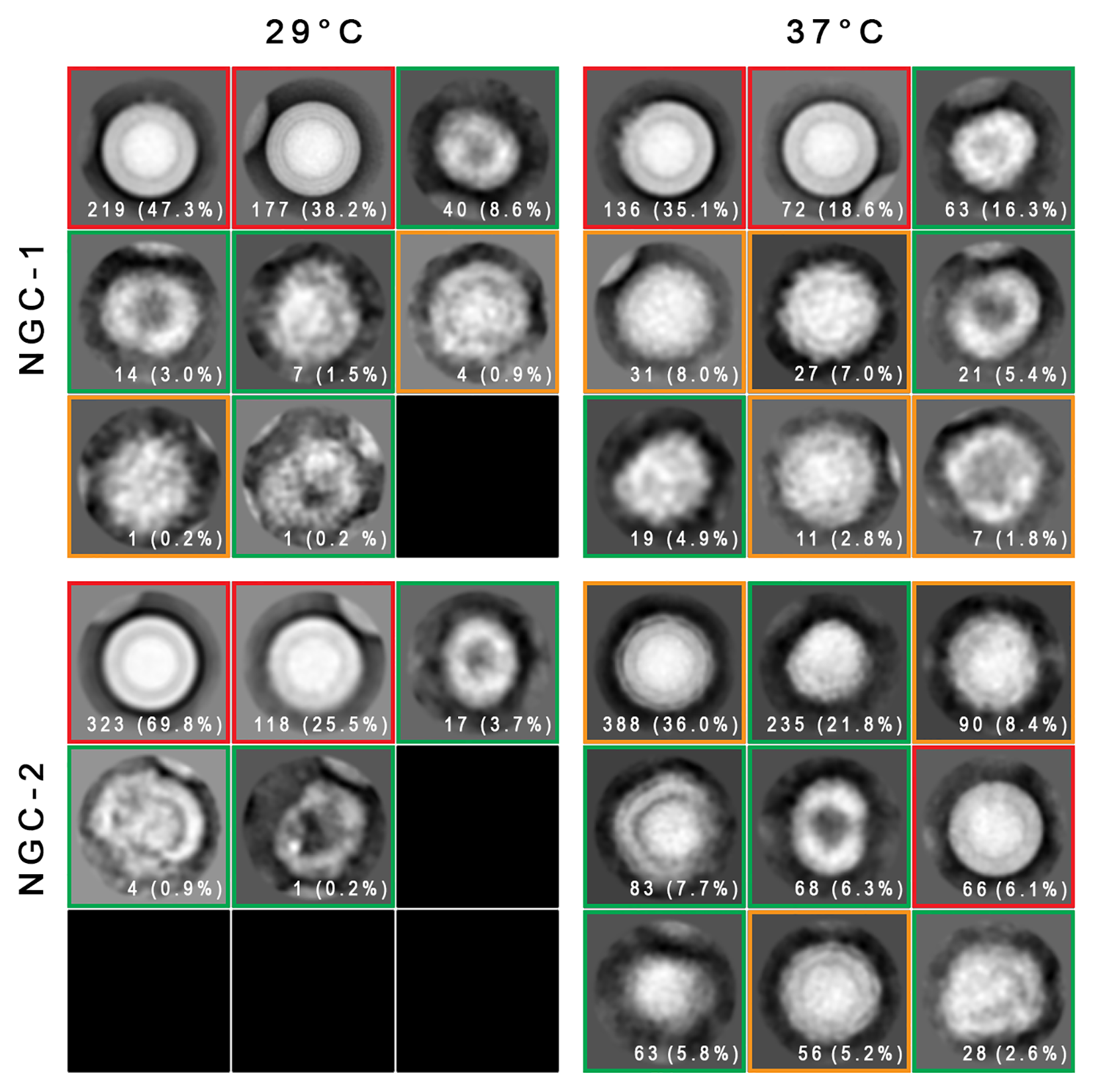

Supplement: S6 Fig — (TIF) [file ppat.1007996.s006.tif]

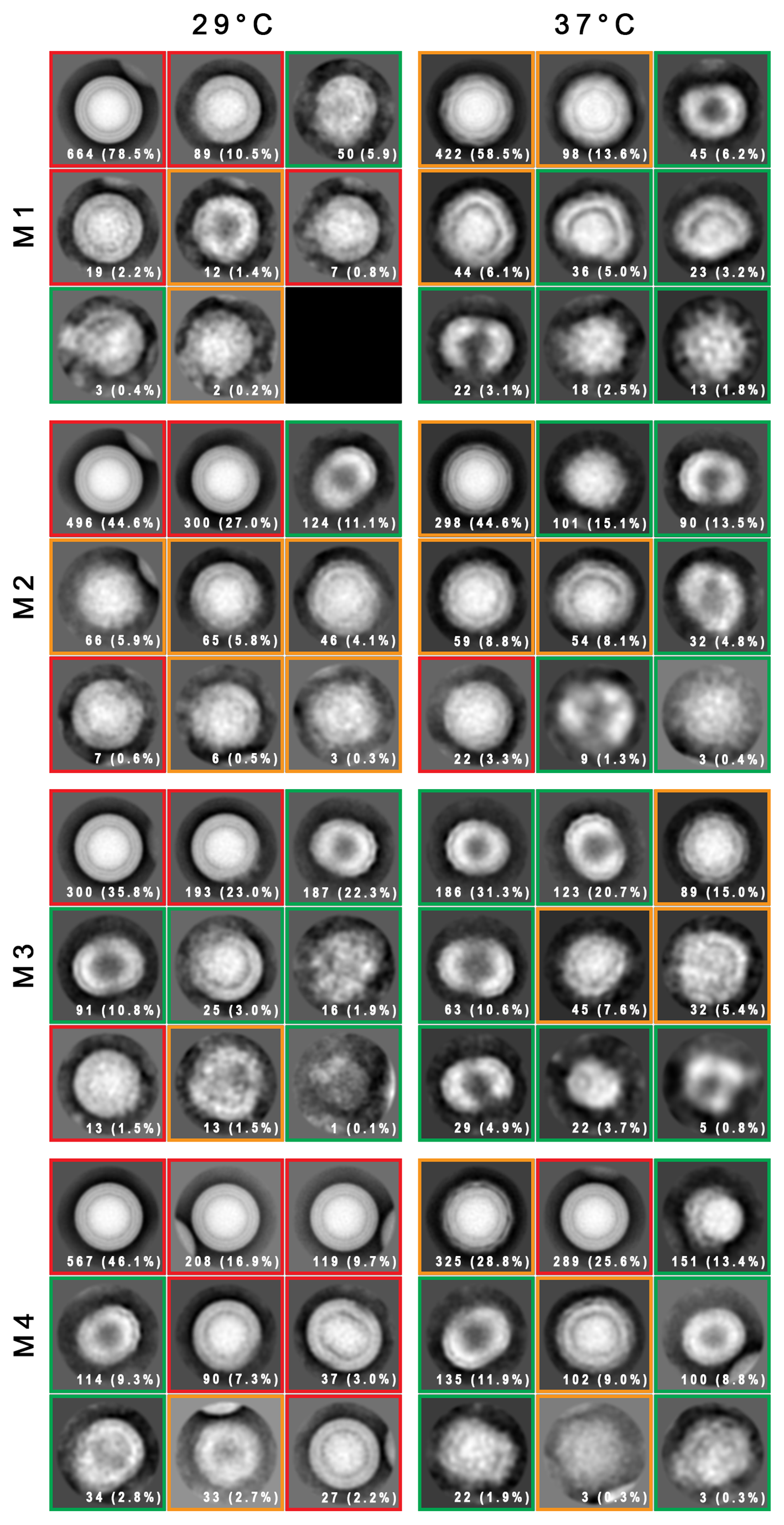

Supplement: S7 Fig — (TIF) [file ppat.1007996.s007.tif]

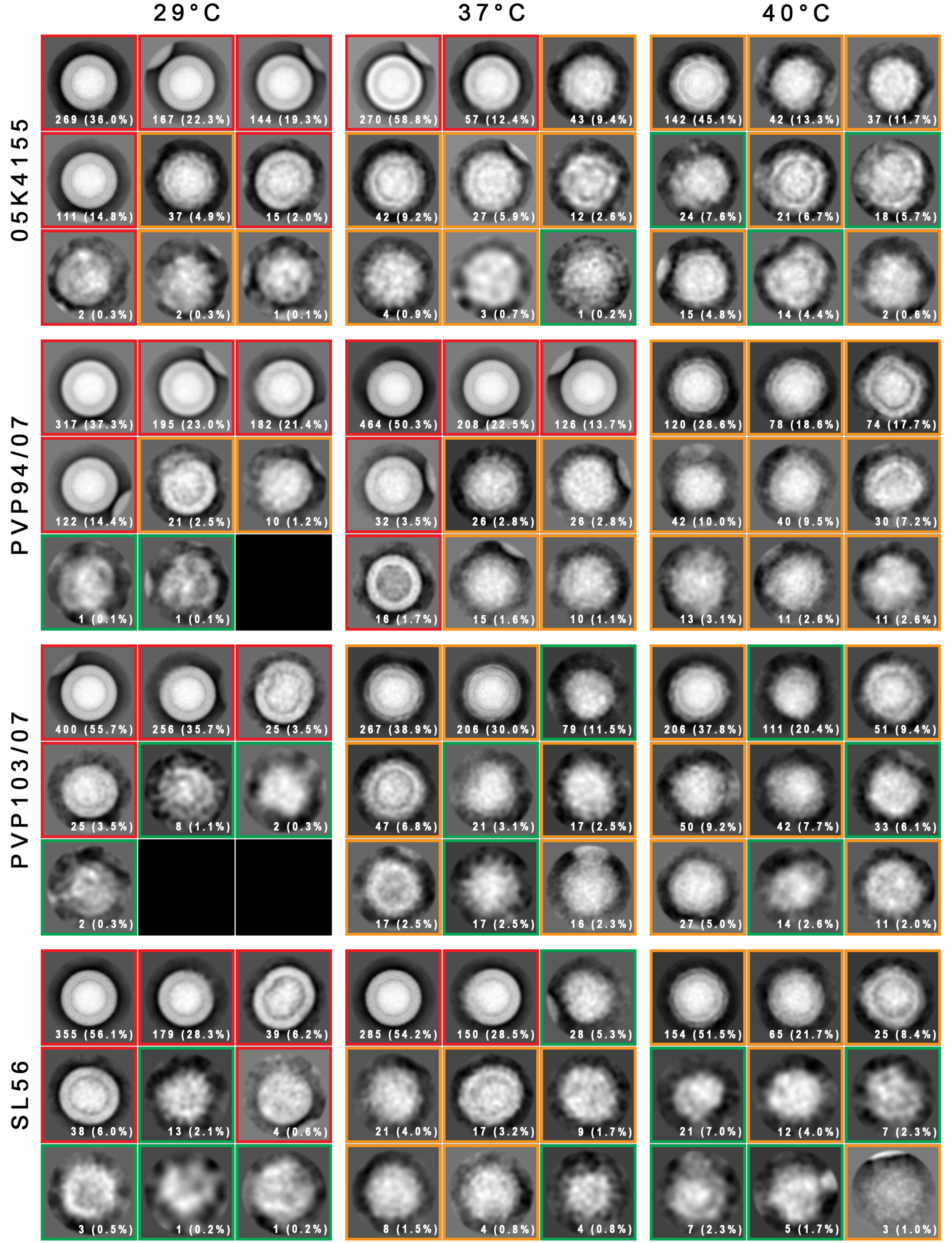

Supplement: S8 Fig — (TIF) [file ppat.1007996.s008.tif]
