# Supplementary material for: Molecular basis of dengue virus serotype 2 morphological switch from 29°C to 37°C
Source: PLoS Pathog. 2019 Sep 19;15(9):e1007996. doi: 10.1371/journal.ppat.1007996 (PMC6752767; doi:10.1371/journal.ppat.1007996)
Supplement: S1 Table — (DOCX) [file ppat.1007996.s013.docx]

S1 Table. Primers for sequencing prME encoded region of DENV2 genome

| **Primer name** | **5’ to 3’ sequence** | **Reference** |
| --- | --- | --- |
| **Forward** | | |
| D2s350_372 | GGAAAGAGATTGGAAGGATGCTG | This work |
| D2s1148_1168 | AATCTCGTTGCCCAACACAAG | This work |
| D2s1792_1823 | AGGCTGAGAATGGACAAACTAC | This work |
| **Reverse** | | |
| D2a21 | CTGAAACCCCTTCTACAAAGTCTC | [38] |
| D2a1669_1690 | CAACAACATCCTGTTTCTTCGC | This work |
| D2a18 | CCACTGCCACATTTCAGTTC | [38] |
