# Supplementary material for: Molecular basis of dengue virus serotype 2 morphological switch from 29°C to 37°C
Source: PLoS Pathog. 2019 Sep 19;15(9):e1007996. doi: 10.1371/journal.ppat.1007996 (PMC6752767; doi:10.1371/journal.ppat.1007996)
Supplement: S2 Table — (DOCX) [file ppat.1007996.s014.docx]

**S2 Table:** Primers and probes for qRT-PCR

| **Primer name** | **5’ to 3’ sequence** | **Reference** |
| --- | --- | --- |
| DENV2_Fw | CAGGCTATGGCACTGTCACGATG | [39] |
| DENV2_Rv | CCATTTGCAGCAACACCATCTC | [39] |
| DENV2_Probe | CTCTCCGAGAACGGGCCTCGACTTCAA | [39] |
| C6/36_b-actin_Fw | CCACCATGTACCCAGGAATC | [40] |
| C6/36_b-actin_Rv | CACCGATCCAGACGGAGTAT | [40] |
| C6/36_b-actin_Probe | CCCCATCCACCATGAAGATCA | This work |
| Hu_b-actin_Fw | TTCTACAATGAGCTGCGTGTGG | [41] |
| Hu_b-actin_Rv | CTGGGGTGTTGAAGGTCTCA | [41] |
| Hu_b-actin_probe | CCCAAGGCCAACCGCGAGAAGAT | [42] |
